# Commercially Available Outbred Mice for Genome-Wide Association Studies

**DOI:** 10.1371/journal.pgen.1001085

**Published:** 2010-09-02

**Authors:** Binnaz Yalcin, Jérôme Nicod, Amarjit Bhomra, Stuart Davidson, James Cleak, Laurent Farinelli, Magne Østerås, Adam Whitley, Wei Yuan, Xiangchao Gan, Martin Goodson, Paul Klenerman, Ansu Satpathy, Diane Mathis, Christophe Benoist, David J. Adams, Richard Mott, Jonathan Flint

**Affiliations:** 1 Wellcome Trust Centre for Human Genetics, University of Oxford, Oxford, United Kingdom; 2 Fasteris, Plan-les-Ouates, Switzerland; 3 Peter Medawar Building for Pathogen Research, Nuffield Department of Medicine, University of Oxford, Oxford, United Kingdom; 4 Section on Immunology and Immunogenetics, Joslin Diabetes Center and Department of Pathology, Harvard Medical School, Boston, Massachusetts, United States of America; 5 Wellcome Trust Sanger Institute, Hinxton, Cambridgeshire, United Kingdom; Stanford University School of Medicine, United States of America

## Abstract

Genome-wide association studies using commercially available outbred mice can detect genes involved in phenotypes of biomedical interest. Useful populations need high-frequency alleles to ensure high power to detect quantitative trait loci (QTLs), low linkage disequilibrium between markers to obtain accurate mapping resolution, and an absence of population structure to prevent false positive associations. We surveyed 66 colonies for inbreeding, genetic diversity, and linkage disequilibrium, and we demonstrate that some have haplotype blocks of less than 100 Kb, enabling gene-level mapping resolution. The same alleles contribute to variation in different colonies, so that when mapping progress stalls in one, another can be used in its stead. Colonies are genetically diverse: 45% of the total genetic variation is attributable to differences between colonies. However, quantitative differences in allele frequencies, rather than the existence of private alleles, are responsible for these population differences. The colonies derive from a limited pool of ancestral haplotypes resembling those found in inbred strains: over 95% of sequence variants segregating in outbred populations are found in inbred strains. Consequently it is possible to impute the sequence of any mouse from a dense SNP map combined with inbred strain sequence data, which opens up the possibility of cataloguing and testing all variants for association, a situation that has so far eluded studies in completely outbred populations. We demonstrate the colonies' potential by identifying a deletion in the promoter of *H2-Ea* as the molecular change that strongly contributes to setting the ratio of CD4+ and CD8+ lymphocytes.

## Author Summary

We show that commercially available mice are a resource for detecting single genes by genome-wide association. We surveyed 66 populations and identified those with properties conducive to high-resolution mapping. Importantly, we show that the same alleles contribute to variation in different colonies, so that when mapping progress stalls in one colony, another can be used in its stead. As a proof of principle, we detect the same QTL in different colonies influencing CD4^+^/CD8^+^ ratios and refine this mapping to the gene level. We show that a deletion in the promoter of *H2-Ea* is the molecular change that strongly contributes to setting the ratio of CD4+ and CD8+ lymphocytes. Our results make it possible for geneticists to make informed choices on the use of colonies for genome-wide association studies of complex traits in mice.

## Introduction

The design of an ideal population for gene mapping involves balancing the avoidance of rare alleles with the requirement for rapid linkage disequilibrium (LD) decay. High rates of LD decay are found in populations with large effective population sizes and many generations of random mating that accumulate recombinants to break up correlations between genotypes. Unfortunately, a necessary corollary is the presence of rare alleles as allele frequencies drift to extremes and new, rare, alleles arise as a consequence of mutations. The more rare alleles in a population, and the more they contribute to phenotypic variation, the more difficult it will be to detect quantitative trait loci (QTLs) using genome-wide association strategies that genotype only common alleles [1].

The best strategy might seem to be to choose animals from highly divergent populations, such as wild mice caught in many locations [2], or from inbred lines derived from highly genetically divergent progenitor strains. This maximizes genetic diversity and seeks to overcome the limitations of using only a subset of the variation present in wild populations. However, mice from different populations will have a high proportion of private variants present in one population only. LD decay for the latter private variants will depend solely on recombinants accumulated during the creation of the colony, while LD decay for the former, common, variants is boosted by the ancestry of the founding populations. Furthermore, the power to detect a genetic effect increases with the minor allele frequency (MAF) of the causal variant. It follows that high power and mapping resolution is best obtained by using animals from the same mating population to reduce the number of private alleles. A related phenomenon is population structure, caused either by recent admixture or uneven degrees of relatedness, both of which should be avoided.

**Figure 1:**
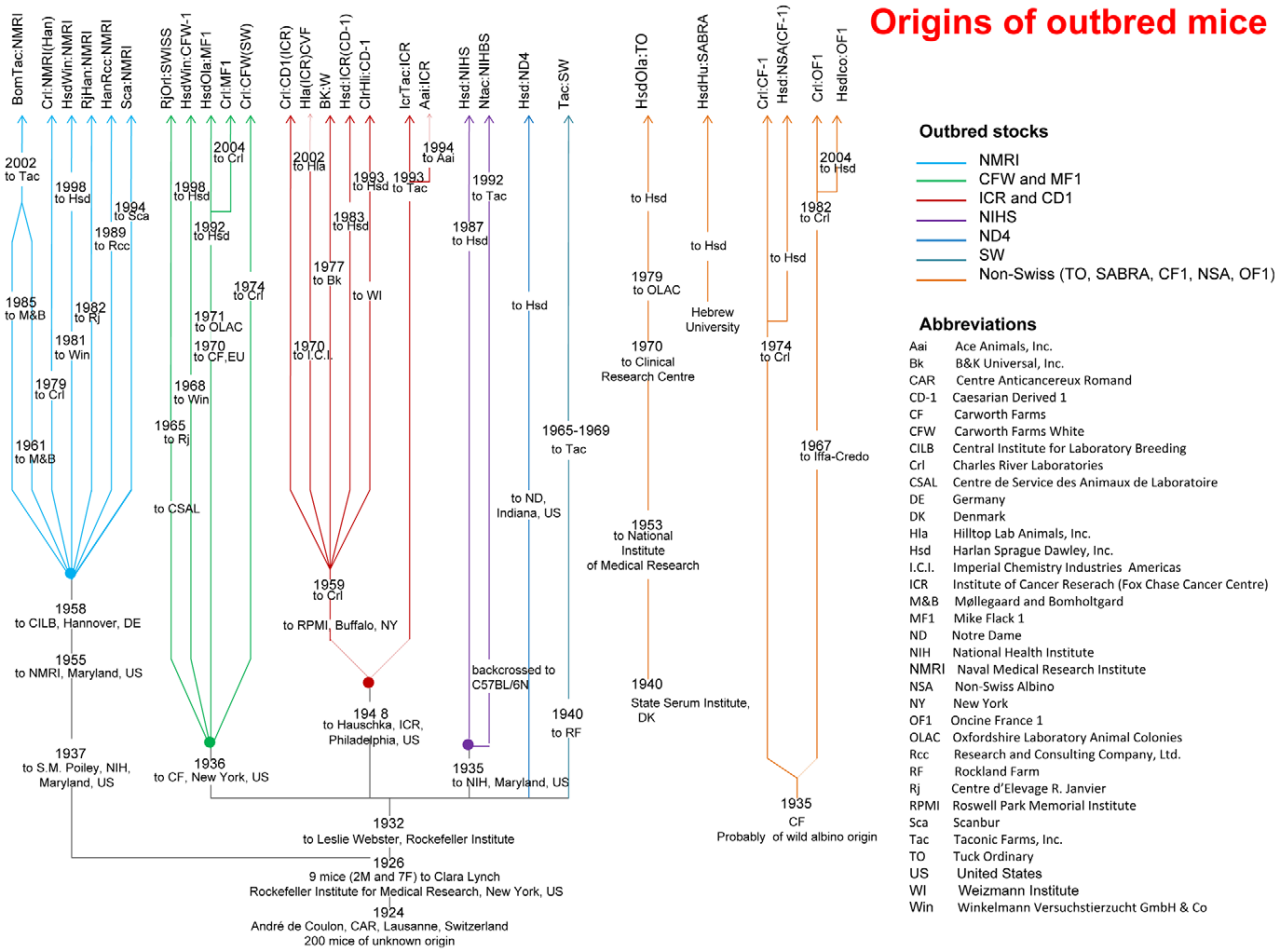
Ancestry of commercially available outbred stocks. Most outbreds have a common origin: they descend from a single Swiss colony of 200 mice from which 2 males and 7 females were imported to the Rockefeller Institute for Medical Research in New York [5]. Outbred Swiss stocks currently available include NMRI, CFW, MF1, CD1, ICR, NIHS, ND4 and SW. Not all outbreds descend from Lausanne in Switzerland. Non-Swiss strains include CF-1, NSA, OF1, SABRA and TO. Details on the origin of colonies are provided in Text S1.

Commercial mouse breeders maintain large colonies of outbred mice that may have the necessary genetic structure. LD in some outbred stocks has been shown to allow high-resolution mapping [3], sufficient to identify genes [4]. Importantly, most outbred stocks are known to derive from animals from a single population, such as the ‘Swiss’ stocks which descend from two males and seven females imported from Lausanne, Switzerland [5], indicating that the proportion of private alleles may be low. Figure 1 summarizes the known relationship between commercially available outbred stocks as of 2007 (the time of this study) and additional information is given in Text S1.

However, without systematic characterization of the genetic architecture of commercially available outbred mice it is not possible to evaluate the suitability of any particular colony for genome-wide association. In this paper we evaluate 66 populations to determine (i) whether inbreeding and population structure preclude the use of the population; (ii) whether linkage disequilibrium (LD) enables high-resolution mapping; (iii) whether the proportions of common and rare variants are favorable for genome-wide association mapping. In order to assess the latter, we tested the hypothesis that the outbred colonies are descended from a common source: the laboratory inbred strains. Populations in which this assumption holds true, and which have low levels of LD, are the most suitable for high-resolution mapping. Finally, we show how commercially available outbred mice can be used to go from genetic association to molecular lesion by identifying a deletion in the promoter of *H2-Ea* as the molecular change that strongly contributes to setting the ratio of CD4+ and CD8+ lymphocytes.

## Results

### Stocks, colonies, and genetic markers

Table 1 lists the populations that we obtained for this study and the numbers of animals we used. We included three control populations, with known genetic characteristics: 12 Heterogeneous Stock (HS) mice [6], 109 Collaborative Cross (CC) mice [7], 94 inbred strains [8] and a population of wild mice caught from multiple sites in Arizona that is likely to represent a fully outbred population, similar to that used in a human genome-wide association study (GWAS) [9].

**Table 1:** Characteristics of outbred mouse colonies.

Colony	No.	Mk.	M/F	Breeding	Gps.	Size	Date	Mean MAF	LD decay radius	Het.	Pct fail HWE	In-breeding coef	Struct.	Status 12/09
Aai:ICR-US	24	351	1/2	Circular	10	1200	24/10/2007	0.026	1.88	0.08	2.27	2.76		
BK:W-UK	48	351	1/6	Poiley	3	925	12/10/2007	0.024	1.12	0.04	2.27	8.78		
BomTac:NMRI-DK_151	23	351	1/3	Poiley	6	453	24/10/2007	0.068	1.07	0.16	1.70	−5.68	Y	*
BomTac:NMRI-DK_160	24	351	1/3	Poiley	6	1038	28/09/2007	0.075	0.87	0.15	1.98	4.57		*
ClrHli:CD1-IL	20	351	1/1	Poiley	4	16	27/11/2007	0.008	2.78	0.01	0.57	−16.50		
Collaborative Cross	109	351					07/11/2008	0.254	NA	0.19	89.24	67.28		
Crl:CD1(ICR)-UK	48	351	7/25	IGS	1	1950	01/08/2007	0.126	1.00	0.27	3.97	4.40	Y	
Crl:CD1(ICR)-US_iso	30	351	1/1	IGS	6	36	10/08/2009	0.152	1.37	0.24	4.25	13.73		
Crl:CD1(ICR)-DE	48	351		Rotational	3	3900	07/11/2008	0.090	1.24	0.19	7.08	10.26		
Crl:CD1(ICR)-FR	48	351					01/12/2008	0.133	0.73	0.28	4.53	6.00		
Crl:CD1(ICR)-IT	48	351	1/4	Rotational		1440	03/11/2008	0.161	0.76	0.31	5.38	4.70		
Crl:CD1(ICR)-US_C61	24	351		IGS	1		10/08/2009	0.114	1.18	0.30	2.27	0.68	Y	
Crl:CD1(ICR)-US_H43	24	351		IGS	1		10/08/2009	0.130	0.89	0.29	3.97	6.00	Y	
Crl:CD1(ICR)-US_H48	24	351		IGS	1		10/08/2009	0.103	1.46	0.30	2.55	−4.18	Y	
Crl:CD1(ICR)-US_K64	48	351	1/3			648	22/11/2007	0.075	0.84	0.30	5.38	−1.41	Y	
Crl:CD1(ICR)-US_K95	24	351		IGS	1		10/08/2009	0.136	1.06	0.28	2.27	−10.45	Y	
Crl:CD1(ICR)-US_P10	24	351		IGS	1		10/08/2009	0.100	1.08	0.22	1.98	1.56	Y	
Crl:CD1(ICR)-US_R16	24	351		IGS	1		10/08/2009	0.085	1.22	0.35	2.83	−12.10	Y	
Crl:CF1-US	48	351	4/15			705	22/11/2007	0.194	2.37	0.35	6.80	10.04		
Crl:CFW(SW)-US_K71	48	351	5/17			350	22/11/2007	0.084	0.86	0.26	4.53	6.28		
Crl:CFW(SW)-US_P08	48	351	1/5			700	25/06/2008	0.068	1.65	0.22	0.00	4.65		
Crl:CFW(SW)-US_P08	20	600K						0.093		0.118	2.03	1.59		
Crl:MF1-UK	47	351	1/1	Non-Sibs	1	30	01/08/2007	0.053	4.06	0.13	1.13	−2.06	Y	
Crl:NMRI(Han)-DE	48	351				5850	07/11/2008	0.128	1.11	0.27	4.82	1.93		
Crl:NMRI(Han)-FR	48	351		Robertson	4	520	25/09/2007	0.139	1.21	0.26	6.23	12.01		
Crl:NMRI(Han)-FR	20	600K						0.111		0.24	4.55	3.14		
Crl:NMRI(Han)-HU	48	351		Random		60	09/12/2008	0.120	1.07	0.26	6.52	0.43	Y	*
Crl:OF1-FR_B22	24	351		Robertson	4	3600	25/09/2007	0.168	2.04	0.35	6.80	−5.27	Y	
Crl:OF1-FR_B41	24	351		Robertson	4	3600	25/09/2007	0.161	2.36	0.35	6.80	−7.98	Y	
Crl:OF1-HU	50	351		Random		72	09/12/2008	0.162	2.27	0.35	6.80	−1.35	Y	*
Crlj:CD1(ICR)-JP	48	351					02/12/2008	0.073	1.34	0.21	7.08	4.61		
HanRcc:NMRI-CH	48	351	1/1	Poiley	12	725	15/11/2007	0.102	1.47	0.20	1.98	−11.67	Y	*
Heterogeneous Stock	12	351						0.207	2.03	0.43	2.83	−3.88		
Hla:(ICR)CVF-US	48	351	1/3	Random	1	2500	26/10/2007	0.098	0.79	0.21	4.82	−3.13		
Hsd:ICR(CD-1)-DE	53	351	1/2	Random	1	750	03/11/2008	0.153	1.08	0.29	5.10	2.13	Y	*
Hsd:ICR(CD-1)-ES	48	351	1/3	Random	1	1563	14/11/2007	0.147	1.49	0.26	5.38	3.49		*
Hsd:ICR(CD-1)-FR	64	351	1/2	Random	1	2000	06/08/2007	0.155	0.99	0.28	5.38	5.60	Y	*
Hsd:ICR(CD-1)-FR	20	600K						0.105		0.22	1.53	−1.82		
Hsd:ICR(CD-1)-IL	48	351	1/3	Random	1	500	27/11/2007	0.143	1.34	0.29	3.68	−6.55		
Hsd:ICR(CD-1)-IT	48	351	1/3	Random	1	1450	16/11/2007	0.162	1.07	0.28	4.82	7.52		
Hsd:ICR(CD-1)-MX	48	351	1/3	Rotational	2	800	04/08/2008	0.153	1.07	0.30	13.60	−11.34		
Hsd:ICR(CD-1)-UK	48	351	1/3	Random	1	500	05/12/2008	0.147	1.24	0.28	3.97	−0.34		
Hsd:ICR(CD-1)-US	48	351	1/2	Random	1	2592	04/08/2008	0.149	1.05	0.28	5.38	4.36		
Hsd:ND4-US	48	351	1/3	Random	1	1002	04/08/2008	0.036	1.79	0.07	2.27	4.89		
Hsd:NIHS-UK_C	15	351	1/3	Random	1	66	05/12/2008	0.055	1.02	0.11	1.70	6.36		
Hsd:NIHS-UK_G	33	351	1/3	Random	1	33	05/12/2008	0.084	2.04	0.11	3.12	−5.09		*
Hsd:NIHS-US	48	351	1/2	Random	1	1163	04/08/2008	0.011	2.45	0.19	9.92	−18.01		
Hsd:NIHSBC-IL	12	351	1/2	Poiley	4	16	27/11/2007	0.047	1.05	0.02	0.57	3.11		*
Hsd:NSA(CF1)-US	48	351	1/3	Random	1	6048	04/08/2008	0.160	1.30	0.34	11.61	1.90	Y	
HsdHu:SABRA-IL	48	351	1/2	Random	1	100	27/11/2007	0.146	2.55	0.22	22.38	25.44		
HsdIco:OF1-IT	48	351	1/2	Random	1	3012	16/11/2007	0.187	1.82	0.34	13.60	5.22		
HsdOla:MF1-IL	8	351	1/2	Poiley	4	16	27/11/2007	0.141	3.38	0.21	1.70	21.38		*
HsdOla:MF1-UK_G	56	351	1/2	Random	1	8544	06/08/2007	0.132	3.14	0.28	3.40	−0.65		
HsdOla:MF1-UK_C	184	351	1/3	Random	1	1837	01/06/2007	0.132	3.18	0.21	4.25	5.31		
HsdOla:MF1-US_202Aiso	24	351					04/08/2008	0.061	0.53	0.13	0.85	−6.90		
HsdOla:MF1-US_202Aprod	24	351	1/3	Random	1	201	04/08/2008	0.061	2.38	0.13	0.85	−9.21		
HsdOla:TO-UK	48	351	1/3	Random	1	420	29/11/2007	0.049	2.84	0.10	3.68	9.47		
HsdWin:CFW1-DE	48	351	1/4	Random	1	460	14/11/2007	0.127	1.51	0.24	7.93	−0.88		*
HsdWin:CFW1-NL	48	351	1/2	Random	1	100	26/11/2007	0.112	0.89	0.21	4.82	3.62		
HsdWin:CFW1-NL	20	170K						0.498		0.18	7.15	2.70		
HsdWin:NMIR-UK	32	351	1/1	Random	1	80	05/12/2008	0.049	1.51	0.12	1.70	−4.89	Y	
HsdWin:NMRI-DE	48	351	1/4	Random	1	1000	26/11/2007	0.098	1.10	0.20	2.27	−8.87		*
HsdWin:NMRI-NL	64	351	1/3	Random	1	999	06/08/2007	0.099	1.04	0.19	3.12	2.11		
HsdWin:NMRI-NL	26	170K						0.045		0.13	7.23	−1.33		
IcrTac:ICR-US	36	351	1/3	Poiley	6	2056	26/10/2007	0.013	1.92	0.06	2.55	5.40		
Inbreds_94_strains	94	351					07/11/2008	0.326	2.32	0.00	98.58	100.00		
NTac:NIHBS-US	36	351	1/3	Poiley	6	440	26/10/2007	0.003	NA	0.01	0.57	−53.44		*
RjHan:NMRI-FR	48	351	4/4	Robertson	4	2400	18/10/2007	0.132	1.00	0.28	13.60	17.80		
RjHan:NMRI-FR	20	170K						0.047		0.18	7.59	10.62		
RjOrl:Swiss-FR	48	351	4/4	Robertson	4	1400	18/10/2007	0.078	0.88	0.17	3.40	−9.22		
Sca:NMRI-SE_22	24	351	1/1	Random	4	100	08/11/2007	0.047	1.09	0.09	3.12	15.16		*
Sca:NMRI-SE_10an	24	351	1/1	Random	4	260	08/11/2007	0.054	1.10	0.09	5.38	22.31		
Sim:(SW)fBR-US_A1	48	351	1/5	Random	3	1800	08/11/2007	0.056	3.02	0.10	3.68	12.43		
Sim:(SW)fBR-US_B1	24	351	1/5	Random	3	700	08/11/2007	0.050	3.05	0.11	1.13	−7.87		
Tac:SW-US	36	351	1/3	Poiley	6	4000	26/10/2007	0.159	1.30	0.33	3.97	−2.00		*
Wild_Arizona	96	351					07/11/2008	0.169	0.38	0.26	38.81	27.86		

For each colony listed in column one, we report the number of animals we used (No.); the number of markers analysed (genome-wide marker sets were used for six populations); the sex ratio (Male to Female); the supplier's breeding scheme (where available); the number of groups of animals used in that scheme and the size of the colony. Some colonies have been culled since the sampling date and these are indicated by a * in the column headed Status 12/09. We provide four genetic measures for each colony: the mean minor allele frequency (Mean MAF), heterozygosity (Het) and an inbreeding coefficient. The LD decay radius, a measure of the colony's suitability for high-resolution mapping, is the average physical separation between SNPs beyond which the squared correlation coefficient drops below 0.5. Colonies where we found evidence for population structure are show in the column Struct. The multi-dimensional scaling of IBS pairwise distance matrices on which this is based is shown in Figure S1.

We use the term “colony” to mean a population of mice maintained as a mating population at a single location, and “stock” to mean a collection of colonies that are given the same stock designation by the breeders. For example, HsdWin:CFW-1 and Crl:CFW(SW) are two colonies from the same stock (CFW). We follow the nomenclature for outbred stocks [10], but add a two letter code for the country of origin and, when there are several cohorts from the same site, a code for the production room: e.g. Crl:CFW(SW)-US_P08.

We analyzed all colonies with 351 markers at two loci on chromosome 1 (131.6–134.5 Mb and 172.6–177.2 Mb), one locus on chromosome 4 (136.2–139 Mb), and one locus on chromosome 17 (32.6–38.9 Mb) (marker details are given in Table S1). The loci were chosen because they include large effect QTLs detected in a mapping study in Heterogeneous Stock (HS) mice [6] that are easy and inexpensive to phenotype (large effect QTLs, explaining more than 10% of the phenotypic variance, can be mapped with about 200 outbred animals). The QTLs were for serum alkaline phosphatase (ALP) on chromosome 4, the ratio of CD4^+^ to CD8^+^ T-lymphocytes on chromosome 17, concentration of high-density lipoproteins (HDL) in serum on chromosome 1, and mean red cell volume also on chromosome 1. The region on chromosome 17 includes the MHC, highly polymorphic in wild populations and a sensitive indicator therefore of any loss of heterozygosity. While these four loci constitute less than 1% of the genome, if QTLs cannot be mapped at high-resolution here, it is unlikely that colonies will be suitable for genome-wide mapping (we also carried out genome-wide analyses in a subset of animals to test this assumption). SNPs at the four loci were spaced so as to allow us to make inferences about both long and short range LD. We assessed the extent of inbreeding and population structure, genetic drift over time, linkage disequilibrium, the proportions of common and rare variants and the extent of genetic differentiation between colonies. Each factor influences the value of a colony for genetic mapping.

### Inbreeding and population structure

High rates of inbreeding make colonies less suitable for mapping because they contain fewer segregating QTLs. Table 1 gives four measures of inbreeding: mean minor allele frequency (MAF), heterozygosity (inbred colonies will score low on this measure); the percentage of markers that failed a test of Hardy Weinberg equilibrium (HWE) [11] (colonies that consist of inbred but unrelated individuals, will have high scores) and a coefficient of inbreeding that compares the observed versus expected number of homozygous genotypes [12].

Four colonies are almost inbred (with heterozygosities less than 5%): NTac:NIHBS-US, ClrHli:CD1-IL, Hsd:NIHSBC-IL, BK:W-UK. A further five colonies have heterozygosities between 5% and 10% and so are unlikely to be useful for mapping. Three colonies have inbreeding coefficients greater than 20% (HsdHu:SABRA-IL, Sca:NMRI-SE_10an, HsdOla:MF1-IL) and a further seven have values greater than 10% (Table 1). None of these colonies are suitable for genetic association studies.

Colonies that consist of a mixture of relatives (such as siblings, half siblings, cousins, second degree and third degree relatives) will be difficult to use for mapping because the differing degrees of genetic relatedness introduce population structure. We looked for evidence of this using multi-dimensional scaling of identity by state (IBS) pairwise distance matrices [13]. Overall, we found two or more clusters in nineteen populations (marked as such in Table 1) (results for all populations are shown in Figure S1). However, while we can observe gross population structure at the markers tested, our power to detect more subtle effects is limited as accurate determination with Fst less than 0.01 requires more than 20,000 markers [14] (Fst is a measure of genetic diversity within and among populations [15]).

We carried out genome-wide analyses in six colonies judged to be most suitable based on the 351 SNP analysis (sparse set of SNPs). Three populations were genotyped using the 600K Affymetrix Mouse Diversity Array [16]. Three more populations were analyzed using a precursor to this array that, after removing poorly performing markers, gave approximately 170,000 genotypes. Results are given in Table 1. To compare results from the high-density arrays to those obtained from using 351 markers, we made 1,000 random samples of 351 markers from the dense marker sets (from four regions matched in size), measuring heterozygosity and inbreeding in each sample. Using the samples we calculated the distribution of each statistic. The mean of the distribution coincided with the value obtained from the whole genome analysis. We then found the percentile position on this distribution of the results we obtained from the 351 markers. In all cases, the results lay within 10% of the distribution mean, indicating that results from our sparse marker set are representative.

### Genetic drift

One potential concern surrounding the use of outbred colonies is that their genetic constitution is not stable and will fluctuate over time, due to unintended directional selection and random genetic drift. Table 1 demonstrates that most colonies are maintained with population sizes of many thousands, which should reduce the effects of shifting allele frequencies. We tested whether this was so by re-sampling six colonies at least one year after our initial analysis and in five cases found good agreement between heterozygosity, relatedness, and inbreeding measured on the two occasions (Table 2). In one case we noted a change in genetic structure. Results obtained from HsdOla:MF1-UK animals used in 2003 were different from those purchased in 2007: heterozygosity fell from 30% to 5% and the inbreeding coefficient rose from 3 to more than 30. Due to infection, the colony had been reformed from a small number of re-derived founders, thereby introducing a severe population bottleneck and explaining the changes in genetic architecture. However, such drastic changes are unusual, are known to the breeders and can be ascertained in advance.

**Table 2:** Temporal variation.

Population	Month	Year	No.	Het.	Pct MAF<5%	Pct fail HWE	Mean inbreeding coef
Crl:CD1(ICR)-US_K64	Nov	2007	48	0.300	14.16	5.38	−1.41
	Aug	2009	24	0.322	4.25	1.98	−5.33
Crl:CFW(SW)-US_P08	June	2008	206	0.216	24.36	0.00	4.65
	Oct	2009	36	0.254	11.33	2.83	−5.29
HsdIco:OF1-IT	Nov	2007	48	0.343	2.27	1.36	5.22
	Feb	2008	48	0.357	9.63	9.07	−3.73
HsdOla:MF1-UK	Mar	2003	52	0.297	2.27	1.98	3.34
	Aug	2007	192	0.051	1.98	31.16	31.20
HsdWin:CFW1-NL	Nov	2007	48	0.205	7.93	4.82	3.62
	Aug	2008	234	0.204	12.18	0.00	10.19
HsdWin:NMRI-NL	Aug	2007	64	0.191	5.95	3.12	2.11
	Aug	2008	200	0.190	8.50	0.00	0.29

### Linkage disequilibrium

**Figure 2:**
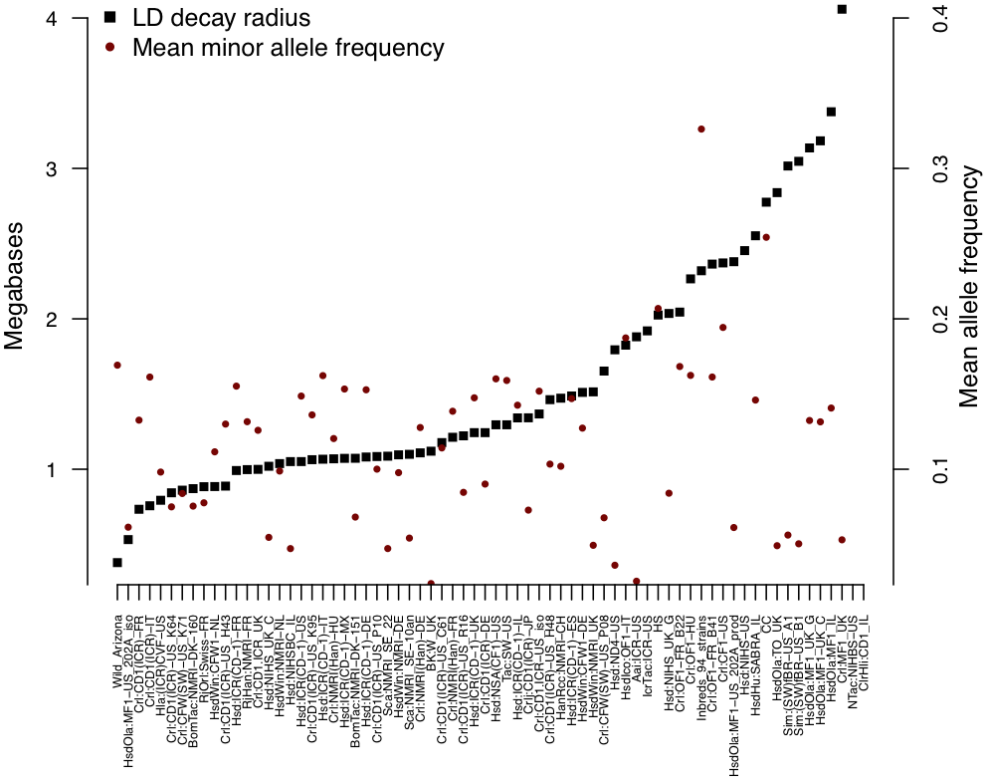
Linkage disequilibrium decay radius (black) and minor allele frequencies (red) in outbred mice. The scale of the vertical axis is megabases for the decay radius and ten times the value of the mean allele frequency (so a value of 2 is 0.2).

Low LD is a requirement for high-resolution mapping. We assessed resolution at the four test loci by the LD decay radius, defined as the average physical separation in base pairs between SNPs beyond which the average squared correlation coefficient (r^2^) drops below 0.5 (Table 1). Figure 2 shows results for all populations analysed (there were insufficient polymorphic SNPs to calculate LD for NTac:NIHBS-US and ClrHli:CD1-IL).

Average figures of LD decay mask variation between regions. For example HsdWin:NMRI-NL has a mean LD decay radius of just over 1, but it will be of little use mapping the MHC region where LD is extensive. However, a region with high LD in one population may have low LD in another. This locus-to-locus variation means that no single population is ideal and that colony-specific genome-wide haplotype and recombination maps are needed.

We explored genome-wide variation in LD in three colonies analysed with the 600K Mouse Diversity Array [16]: Crl:CFW(SW)-US_P08, Crl:NMRI(Han)-FR and Hsd:ICR(CD1)-FR. Mean block length varied between the three colonies: Crl:CFW(SW)-US_P08 79.2 Kb (standard deviation (sd) 70.8), Crl:NMRI(Han)-FR 39.53 Kb (sd 58.7), and Hsd:ICR(CD1)-FR 51.1 Kb (sd 79.5). Block data for each chromosome is given at http://www.well.ox.ac.uk/flint-old/outbreds.shtml. Since there is on average about one gene per 100 Kb, gene-level resolution mapping is possible in these three colonies.

### Haplotypes in commercial outbreds are found in laboratory strains

**Figure 3:**
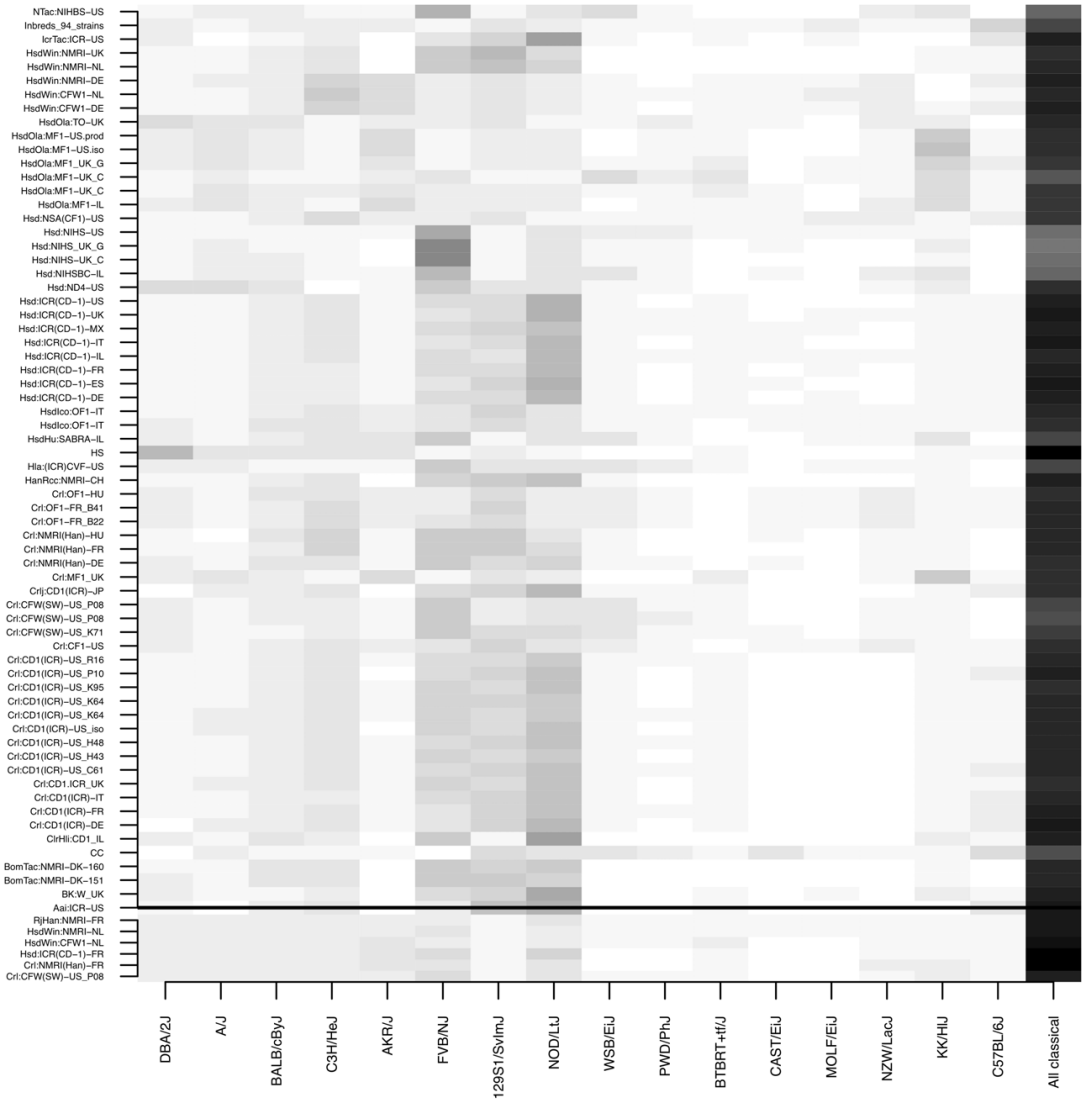
Proportion of laboratory inbred strain haplotypes found in commercial outbred stocks. The region above the horizontal black line gives results from an analysis based on 351 markers from four regions in 66 colonies. Below the black line are results from a genome-wide analysis of 6 stocks. The degree of grey scale represents the contribution from each of the Perlegen re-sequenced strains [18] to the outbred colonies.

Genome-wide association will be effective in colonies where all, or the majority of haplotypes are tagged by markers on a high-density array. The colonies' ancestry, as depicted in Figure 1, suggests they contain a relatively limited set of haplotypes, present in inbred strains. We estimated the contribution of each inbred strain to each colony's genetic architecture by reconstructing the genome of each mouse as a probabilistic mosaic of the founders [17]. We used the Perlegen NIEHS genotypes [18] from 15 inbred strains and analysed all colonies at the four loci (Figure 3) and performed genome-wide analyses in six colonies.

While there is considerable variation between colonies, two general patterns are clear in both locus-specific and genome-wide analyses. First, in all colonies, the fraction of haplotypes accounted for by classical inbred strains ranges between 42% (the NIHS colonies) to 80% (most ICR/CD1). Second, the wild-derived strains (WSB, CAST, MOLF) contribute the least (3–5%). The NIHS stocks contain the highest contribution of the Swiss mouse FVB (25–35%). NMRI are 15–20% FVB and 15% 129, CD1 about 15% FVB and MF1 only 5%. The CFW stocks all contain about 15% FVB. The genome-wide results are similar, except the overall contribution of 129 is closer to the other classical inbred strains. These results confirm that haplotypes in outbred colonies are predominantly the same as those found in classical laboratory inbred strains and suggest outbred stocks originated from mice genetically similar to inbred strains.

### Sequence analysis and novel variants

The haplotype analysis might be subject to SNP ascertainment bias as only variants segregating among inbred strains were genotyped. Furthermore, ancestral haplotype reconstruction always finds representations of the outbreds' genomes as mosaics of a given set of inbreds; it does not test if the ancestral hypothesis is true in general, nor whether the set of founders is optimal in the sense of explaining the genome structure of outbred mice with the fewest recombinants and inbred strains. However, the ancestral hypothesis would be refuted if many SNPs segregated within the stocks that are not found in inbred strains. Colonies with high rates of these private alleles will be less suitable for genome-wide association studies.

We assessed how many SNPs, missing in laboratory inbred strains, are present in the outbred colonies. We amplified and sequenced 22 fragments of about 1.2 Kb, from eight regions in a 5 Mb region previously sequenced on mouse chromosome 1 [19] and from a further 14 regions within the four QTLs described above. We sequenced 12 animals from three populations (HsdWin:CFW-NL, Crl:CFW(SW)-US_K71 and HsdWin:NMRI-NL), 12 wild mice (DNA provided to us by François Bonhomme, University of Montpellier) and 10 classical inbred strains (A/J, AKR/J, BALB/cJ, C3H/HeJ, C57BL/6J, CBA/J, DBA/2J, LP/J, I/LnJ and RIII/DmMobJ).

We identified 120 SNPs (Table S2). Wild mice have an average of one SNP every 200 bp but this rate varies between colonies: HsdWin:CFW-NL and Crl:CFW(SW)-US_K71 have a frequency of one SNP every 350 bp, whereas HsdWin:NMRI-NL has one SNP every 520 bp. We compared this set with SNPs detected by whole genome re-sequencing of 13 inbred strains that are not wild-derived (129P2, 129S1/SvImJ, 129S5, A/J, AKR/J, BALBc/J, C3H/HeJ, C57BL/6N, CBA/J, DBA/2J, LP/J, NOD and NZO http://www.sanger.ac.uk/resources/mouse/genomes/). We found three novel variants (rate 2.5%) in Crl:CFW(SW)-US_K71 and only one in HsdWin:CFW-NL and HsdWin:NMRI-NL (rate 0.8%). The low fraction of novel SNPs suggests that known inbred strains can account for most of the genetic variation in the colonies tested.

We took two approaches to determine whether these locus-specific results were representative of the rates of SNPs across the genome. First, we made a single library from four mice from the Crl:CFW(SW)-US_P08 colony, and sequenced sufficient short reads (∼100 bp) to cover the complete genome at ten-fold coverage. We mapped all reads to the reference genome using MAQ, called SNPs using SAMtools[20], [21] and identified a high confidence set of 2,554,879 SNPs. We again compared SNPs with the 13 inbred strains and found that 3.2% of the Crl:CFW(SW)-US_P08 SNPs were novel.

In the second approach, we sequenced libraries of reduced complexity from pooled DNA samples, obtaining high coverage of a small fraction of the genome (∼2%). We validated the method by comparing the rate of novel variants found among 36,154 SNPs from a Crl:CFW(SW)-US_P08 reduced-representation library to the rate obtained from our whole-genome sequence described above. 11.7% of the SNPs in the Crl:CFW(SW)-US_P08 sequence were novel. Since the false discovery rate is estimated to be 8% [22], this result implies a novel SNP rate of approximately 4%, consistent with the finding from the whole-genome sequence. We examined four animals from HsdWin:CFW-NL and HsdWin:NMRI-NL colonies and identified 4,885 and 16,724 SNPs respectively. 3.1% of SNPs from HsdWin:CFW-NL and 5.7% of SNPs from HsdWin:NMRI-NL were unique (i.e. not found in the set of SNPs from the inbred strains). These percentages are consistent with there being few, or no novel SNPs in the HsdWin:CFW-NL and HsdWin:NMRI-NL colonies.

Results from genome-wide sequence thus support the conclusions of the locus-specific results: about 95% of the polymorphisms in the colonies are derived from classical inbred strains (those not derived from wild mice). This result is likely true for other colonies, meaning they contain a relatively limited set of haplotypes, consistent with the reconstructions of each mouse as a mosaic of inbred founders described above (Figure 3).

### Genetic differentiation between colonies

Our genetic characterization of outbred colonies implies that while the same QTL alleles will segregate in different colonies, their frequencies may vary substantially, so that a QTL segregating in one colony may not be detectable in a second. We assessed the extent of genetic differentiation between colonies and stocks using principal components and Fst distances. We found extensive population differentiation: Fst between populations is 0.454.

**Figure 4:**
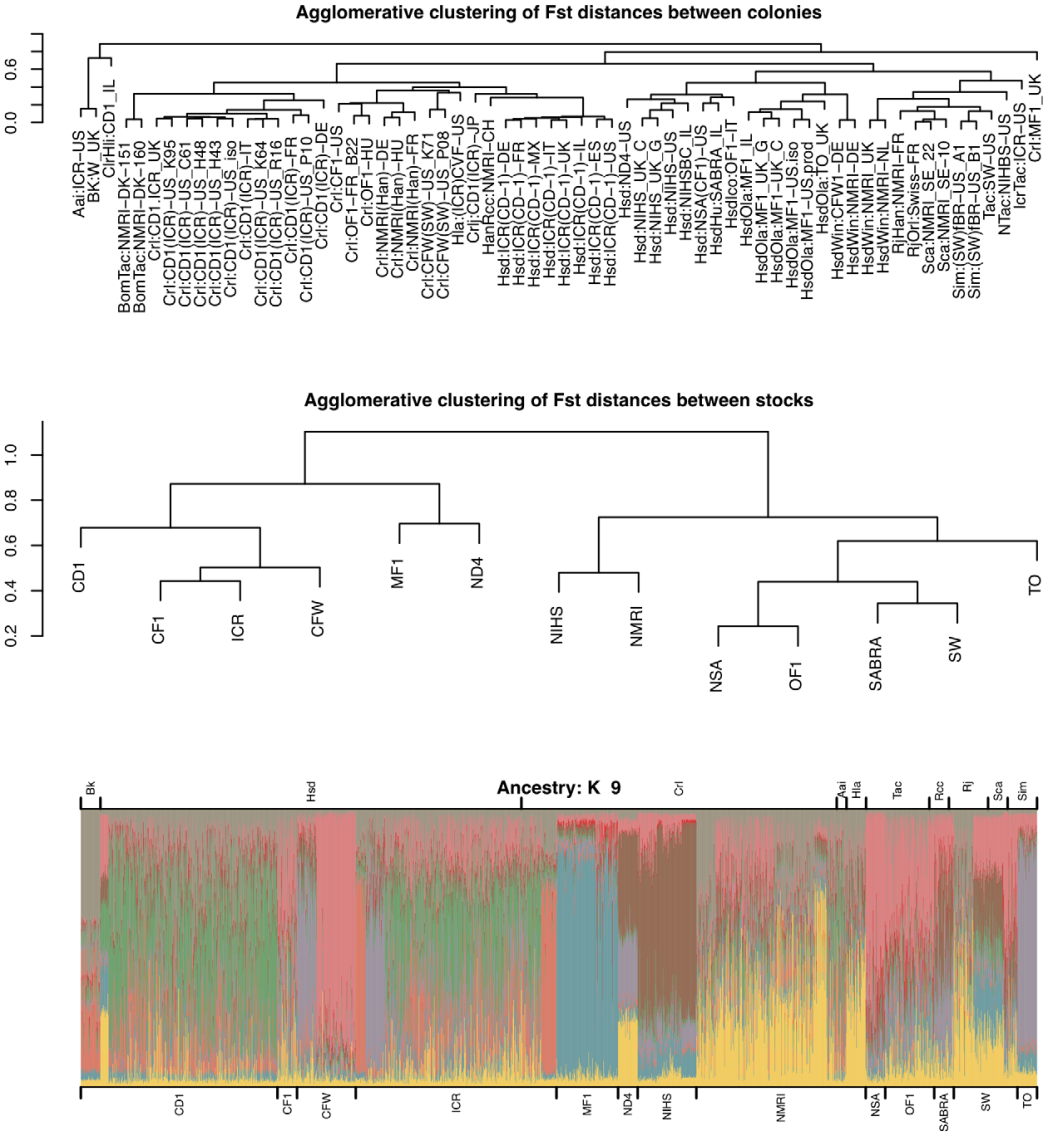
Colonies, stocks, and ancestry. Top two panels: relationship between colonies (top) and stocks (middle panel) shown by agglomerative clustering of Fst distances. Bottom panel: ancestry inferred from the FRAPPE program at K = 9. The length of each colored corresponds to the ancestry coefficient of each mouse, plotted along the horizontal axis. Mice are labeled by stock name (along the bottom) and by commercial provider along the top. Mice of the same colony were grouped together (giving rise to blocks of common ancestry, as seen for example to the right of the CD1 cluster) but individual colony labels omitted.

No single feature, not stock, colony, producer or country of origin, satisfactorily accounted for genetic differentiation. The top panel of Figure 4 shows the relationships between colonies and the middle panel the relationship between stocks (Figure S2 shows similar results obtained by principal components). We then characterized genetic relationships between colonies regardless of stock identity, using methodologies established in studies of human populations: we considered each colony as originating from K unknown ancestral populations and looked at values of K from 2 to 12 using a maximum likelihood method in the program FRAPPE [23], [24]. Figure 4 (bottom panel) shows the results for K = 9 (see http://www.well.ox.ac.uk/flint-old/outbreds.shtml for all values of K). At no value of K were we able to differentiate all stocks.

Stocks differ primarily in the proportions of common ancestry, consistent with their descent from inbred strains. There is considerable variation within a stock, largely explained by variation between colonies, as shown for example by CD1 and NMRI stocks. Taken together the dearth of private alleles, we conclude that quantitative differences in allele frequencies are responsible for population differences.

### QTL mapping

From the data in Table 1 we selected three colonies (Crl:CFW(SW)-US_P08, HsdWin:CFW-NL and HsdWin:NMRI-NL) suitable for high-resolution mapping. We mapped four QTLs previously detected in HS mice [6]: serum alkaline phosphatase (ALP) on chromosome 4, the ratio of blood CD4^+^ to CD8^+^ T-lymphocytes on chromosome 17, the concentration of high-density lipoproteins (HDL) in serum on chromosome 1, and mean red cell volume (MCV) on chromosome 1. Since HS mice contain alleles derived from eight inbred strains we expect the QTL alleles also to be present in a proportion of the outbred colonies.

**Figure 5:**
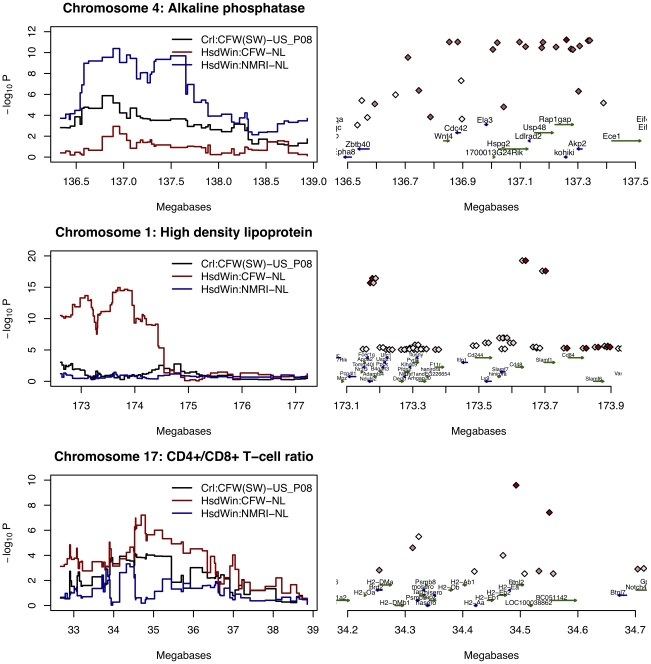
Association mapping of three phenotypes in three colonies. The vertical scale is the negative logarithm (base10) of the P-value for the association; the horizontal scale is the position in megabases on the chromosome. On the left of the figure are results for ancestral haplotype reconstruction analysis (HAPPY) for all three colonies; on the right single marker association is shown for one colony for each phenotype: HsdWin:NMRI-NL for alkaline phosphatase; HsdWin:CFW-NL for high density lipoprotein and Crl:CFW(SW)-US_P08 for the CD4^+^/CD8^+^ T-cell ratio. LD structure around the associated SNP is shown by a red to white scale for r^2^ = 0 to 1. For high density lipoprotein, each marker is represented by two diamonds. The right hand diamond of each pair is colored to show the r^2^ with rs3476237 (at 173.6 Mb and the left hand the r^2^ with rs3709584 (at 173.1 Mb). Gene annotations are taken from the UCSC genome browser.

We tested first whether QTLs could be detected under the assumption that the QTL alleles descend from inbred progenitors. To do so, we used the ancestral haplotype reconstruction described above and mapped QTLs with the HAPPY software package [17]. The detection of QTLs differed markedly between colonies. There was no evidence for association between any markers on chromosome 1 influencing MCV in any colony (data not shown); single marker association also failed to detect an effect for this phenotype. However, probabilistic ancestral haplotype reconstruction was successful in detecting QTLs for the other three phenotypes. By permutation, we obtained region-specific 5% significance thresholds for HsdWin:NMRI-NL, HsdWin:CFW-NL and Crl:CFW(SW)-US_P08 respectively for ALP of 2.8, 2.4 and 2.6, for HDL of 2.2, 2.9 and 2.6 and for CD4^+^/CD8^+^ ratio of 3.3, 2.9 and 2.1, here expressed as a negative logarithm (base 10) of the P-value (logP). Results shown in Figure 5 exceed these thresholds for each phenotype, but not in every colony.

The logP for association with ALP exceeded 2.5 for all colonies in a 400 Kb region between 136.9 Mb and 137.3 Mb on chromosome 4 with considerable variation in the strength of association (logP of 11.5 in HsdWin:NMRI-NL and 2.7 in HsdWin:CFW-NL). One colony showed strong evidence for association with HDL (HsdWin:CFW-NL) with a logP>18; two colonies showed association at the chromosome 17 locus with CD4^+^/CD8^+^ T-cell ratio (HsdWin:CFW-NL and Crl:CFW(SW)-US_P08). The percentage of variance explained by each QTL is consistent with effect sizes for these phenotypes found in the HS [6]: 15% for CD4^+^/CD8^+^ T-cell ratio (in Crl:CFW(SW)-US_P08), 11% for HDL (in HsdWin:CFW-NL) and 18% for ALP (in HsdWin:NMRI-NL).

If the QTL alleles are identical in the three colonies, then a single trait effect for each founder strain, independent of colony, should fit the data as well as a model in which each colony had independent effects. This allows us to test, for example, whether the QTL alleles influencing ALP in all three colonies are the same. We found that a model for the single trait effect fitted the data as well as one allowing for independent effect. At the peak of association for ALP the P-value of the partial F test was 0.10; for HDL the P-value was 0.27 and for CD4^+^/CD8^+^ T-cell ratio, 0.92. Our results indicate that the same QTL alleles are present in the different colonies and confirm that QTL mapping is possible on that assumption.

We then assessed QTL mapping resolution. We wanted to know if our predictions of gene-level mapping (based on estimates of haplotype block length) were upheld in practice. HAPPY mapping results, in Figure 5, indicate a region of over 1 Mb likely to contain each QTL. While this is a smaller region than observed in HS outbred mice (where the mean size of QTL intervals is about 3 Mb) it is larger than suggested by the mean LD decay radius (of about 1 Mb). In fact, the size of the QTL interval is deceptive for two reasons: first, we may not have modeled the descent from the correct set of progenitors; second, in the absence of fine-scale recombination data, HAPPY mapping assumes a uniform genetic map, without hotspots, so that the localization is relatively imprecise. We resorted therefore to using single marker analysis and considering the LD structure of each region to determine the most likely position of the QTL (Figure 5).

Analysis of the ALP QTL revealed in all colonies a large region of linkage disequilibrium extending from 136.7 to 137.3 Mb, consequently limiting mapping resolution. The region contains an alkaline phosphatase gene (*Akp2*) at 137.3 Mb, but also an additional 9 genes. Mapping the QTL on chromosome 1 for HDL identified two peaks: rs13476237 at 173631526 and rs3709584 at 173177625 (Figure 5). In the colony showing association (HsdWin:CFW-NL), r^2^ between these markers is low (0.21) and conditioning on the first marker failed to remove the effect attributable to the second (F = 15, df = 2,210, logP = 6.1). These results indicate that two separate effects contribute to the variation in HDL, one co-localizing with *Apoa2*, already known to be involved in this phenotype, and the other over a region containing two genes, *Cd48* and *Slamf1*, neither previously implicated in the regulation of HDL levels.

On chromosome 17, we found a single peak of association for CD4^+^/CD8^+^ ratio at 34.49 Mb (rs33573309). Association with this marker is strongest in the Crl:CFW-US_P08 colony; r^2^ between rs33573309 and rs33699857 at 34550471 is 0.97, but drops to less than 0.3 elsewhere, delimiting a region of 60 Kb containing four genes (Figure 5). Only two of these genes show a strong signal in the joint analysis. *BC051142* (a.k.a. *Tesb*) is a testis-expressed EST of which little is known; on the other hand, *H2-Ea* encodes the alpha chain of the MHC class II Eαβ heterodimer, one of the two complexes which govern the selection and survival of CD4^+^ T cells, and is thus a highly plausible candidate. A number of mouse strains carry a null mutation of *H2-Ea*, most often through a 650 bp deletion in the promoter region [25], [26], and this deletion is tagged by rs33699857.

**Figure 6:**
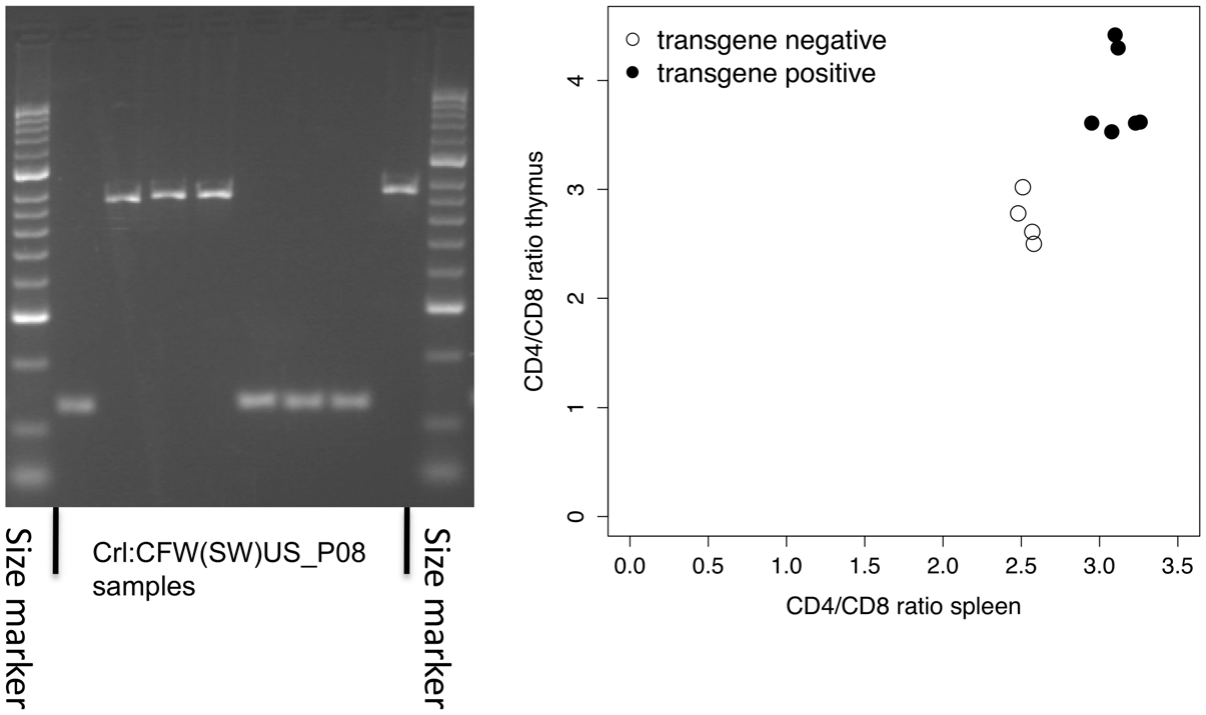
A deletion in the promoter of the alpha chain of the MHC class II Eαβ heterodimer contributes to variation in the ratio of CD4^+^ to CD8^+^ T-lymphocytes. On the left is shown PCR analysis of eight outbred mice demonstrating the presence of the deletion in the colony: smaller bands on the gel indicate animals with a homozygous deletion. On the right is shown the results of complementation with a transgene; the proportion of CD4+ and CD8+ cells was measured by flow cytometry in CD3+ splenocytes and CD3hi single-positive thymocytes of Eanull inbred NOD mice carrying the Ea16 complementing transgene (filled circles) or transgene-negative littermates (open circles).

We confirmed that the promoter deletion is present in the Crl:CFW(SW)-US_P08 by examining reads from the whole-genome sequence lying between 34,485,333 and 34,483,847 bp (http://www.well.ox.ac.uk/flint-old/outbreds.shtml). We designed primers to amplify across the deletion and tested for its presence in mice from HsdWin:NMRI-NL and the Crl:CFW(SW)-US_P08 colonies (Figure 6). We performed a complementation analysis to test *H2-Ea*, measuring CD4^+^/CD8^+^ ratios in mice in which the *H2-Ea* null mutation was complemented by introduction of the Eα16 transgene, which drives normal expression of Ea protein with the normal distribution [27]. As illustrated in Figure 6, the presence of the transgene led to an increased representation of CD4^+^ cells relative to transgene-negative Eanull littermates, confirming the assignment. This increase was present in both the thymus (single-positive mature thymocytes) and spleen, indicating that the variation most likely affects positive selection and lineage commitment of CD4^+^ T cells.

## Discussion

Commercially available outbred mice are used primarily by the pharmaceutical industry for toxicology testing, on the assumption that they model outbred human populations, a view supported by limited genetic surveys [28]. In fact, very little is known about their genetic architecture and assumptions about the combined effects of fluctuating allele frequencies (due to genetic drift) and lack of genetic quality control have led some to argue against their use in genetic investigations [29], [30]. Our catalogue of the genetic structure of commercially available populations makes a systematic evaluation possible for the first time. Our systematic evaluation of their genetic architecture reveals three important features.

First, variation between colonies is large. Fst, a measure of variation within and between populations, is 0.454 (in contrast, human populations values are typically less than 0.05 [31]). The source of this variation is not straightforward. Stock names (such as NMRI or CD1) do not account for it, nor does the supplier, or the country of origin. While some stocks, such as TO and MF1, do indeed have a unique genetic ancestry, many do not. Two likely causes are genetic bottlenecks during colony formation and genetic contamination. Thus, ICR colonies from Harlan and CD-1 colonies from Charles River Laboratories cluster together (Figure 2), having experienced a single bottleneck during their creation (Figure 1). Gene flow appears to have occurred between a number of stocks, as for example between CFW (HsdWin:CFW) and NMRI (HsdWin:NMRI) colonies of Harlan. Both were bred at the Winkelmann Versuchstierzucht GmbH & Co and could easily have been mixed. A similar story probably explains the close genetic relationship between RjHan:NMRI and RjOrl:Swiss.

Apart from breeders' interventions, colony genetic architecture is stable over time. Mouse colonies are often believed to behave very much like finite island populations, so that, except for imposed bottlenecks (as happened with the HsdOla:MF1-UK) or the forcible introduction of new alleles, genetic variation will depend on the effective population size (Ne). Assuming random mating, the time required for a neutral allele to go to fixation in a population, and hence to reduce heterozygosity, is approximately equal to four times Ne. Given that so many colonies are maintained with effective population sizes of many thousands, colony genetic architecture should be stable. Consistent with this view, our analyses of five colonies over two years found little evidence for changes in allele frequencies and LD values.

One important caveat is the introduction by some breeders of systems to maintain heterozygosity by periodically crossing the colony to animals taken from a much smaller population, using a protocol called IGS (International Genetic Standard [32]). In consequence, a small number of chromosomes are distributed widely throughout the population, introducing large regions of linkage disequilibrium that significantly reduce mapping resolution. Colonies subject to this protocol become useless for high-resolution genetic mapping (documented in Table 1).

Second, the number of alleles segregating in colonies is relatively limited (compared to a wild population). Almost all of the genetic variants can be found in classical laboratory strains. Both locus-specific and genome-wide sequencing support this conclusion and haplotype reconstruction demonstrates how variants in the outbreds can be modeled as descending from inbred progenitors.

Third, in terms of mapping resolution, no mouse colony is comparable to a human population. Using an LD criterion, the best mapping resolution in any colony is at least twice that obtainable in human populations. Applying the same definition of a haplotype LD block as used in human LD studies, we found an average block size in three colonies of approximately 60 Kb. By contrast, in African populations average block length is 9 Kb, and 18 Kb in European populations [33].

These observations have important implications for the use of commercial outbreds for genetic mapping. First, the predominance of SNPs from classical inbred strains means that arrays designed using those SNPs, such as the Affymetrix 600K Mouse Diversity Array [16], will capture the majority of genetic variation. Second, the extent of LD means that genome-wide coverage can be obtained with fewer SNP than in highly outbred and genetically heterogeneous populations: using 2 markers to tag each block and assuming an average block size of 50 Kb less than 200,000 markers will capture the majority of the variation in the genome, so the Affymetrix 600K Mouse Diversity Array [16] will be adequate. Third, resolution will fall short of gene level in some regions. But, since LD structure differs between colonies, high resolution mapping of a locus may be possible in one colony, but not in another – no single colony is ideal.

However, mapping resolution is not the only useful measure of a colony's suitability for GWAS. Another critical measure is allele frequency. Large numbers of rare variants contributing to phenotypic variation in a population will make the trait difficult to map using standard GWAS designs. In this regard, our data reveal a favorable situation: QTL mapping, assuming a common set of founder strains, shows that the QTLs replicate between stocks in a consistent manner. These findings indicate that quantitative differences in allele frequencies, rather than the existence of private alleles, are responsible for the population differences. Furthermore, the limited sequence diversity means it is possible to impute the sequence of any commercially available mouse from the sequences of inbred strains. Thus, the full catalogue of sequence variation in a stock could be obtained by sequencing the inbred strains presumed to be founders for it, and genotyping the stock at a skeleton of SNPs. Therefore, we should be able to detect the effect of all variants, a situation that has so far eluded studies in completely outbred populations.

Seen in this light, the relatively high degree of genetic differentiation between colonies becomes an advantage. The various genetic architectures available, with variation in QTL frequencies, LD extent and the position of LD blocks, mean that mapping in multiple populations will enable new strategies for gene identification in complex traits. Importantly, we have shown that, at least in the QTLs examined here, the same alleles contribute to variation in different colonies, so that when mapping progress stalls in one stock, another can be used in its stead.

As a proof of principle, we have demonstrated the advantages of mapping in different colonies by detecting the same QTL influencing CD4^+^/CD8^+^ ratio and were able to refine this mapping to the gene level, transgene complementation helping to establish as the causal change the deletion in the *H2-Ea* promoter, a loss-of-function mutation that has long been fixed and segregates widely in the *Mus* species [26]. A strong genetic influence on the CD4^+^/CD8^+^ ratio in mice and human has long been known, predominantly reflecting the efficacy of positive selection [26]. Since MHC class-II molecules such as Ea condition the thymic selection of CD4^+^ T cells, they are thus highly plausible candidates. The homologous MHC class-II region (HLA-DR) has recently been shown to influence CD4+/CD8+ ratio in human blood [34], providing cross-species validation of our result and an example of how results from mice can inform human genetic studies.

A variety of resources are available for mapping complex traits in mice, each with its own advantages. The choice of which to use depends on the researcher's aims. We advocate commercial outbreds as a resource for finding genes. In some circumstances, as we have shown, it is possible to go from genetic association to a gene in a single step. The sequence variants in commercial outbred colonies are almost solely those present in classical laboratory strains, resulting in three advantages. First, it provides low LD: the colonies do not depend on recombinants accumulated since their foundation. Second, the relatively low genetic diversity increases power to detect a QTL, provided that it segregates, because there will be fewer QTLs overall. Simply put, in a population with ten variants the relative contribution of each is ten times the contribution of each locus in a population with 100 variants. Third, phenotypes known to show heritable variation among the classical laboratory inbred strains will show heritable variation within the outbred colonies.

However, the relatively limited genetic diversity of the outbred colonies means that they do not model a fully outbred population; nor can they be used to assess the effect of all variants present in mouse populations. The colonies contain a relatively small subset of that variation. They are likely to have “blindspots” where little functional variation segregates. The creation of the Collaborative Cross (CC), a large set of recombinant inbred lines derived from genetically diverse progenitors [7], provides access to a more complete catalog of variation [35], and also has the advantage of allowing researchers to interrogate the same genotype multiple times and hence accumulate an increasingly rich understanding of the relationship between genotype and phenotype. However, it is not yet clear to what extent CC animals will provide high-level mapping resolution, although simulations suggest it will be of the order of 1–2 Mb [36].

Assuming an investigator decides to use an outbred colony, which is the best to choose? For single locus assays, for example attempting to refine a locus identified in a cross between two inbred strains, the choice will depend on whether the appropriate alleles are segregating at the locus, and this can be assessed by haplotype reconstruction from genotype data. The extent of genetic diversity between colonies (ten times that between different human populations) indicates that an appropriate colony will be found. However, genome-wide data will be needed from all colonies to enable a comprehensive assessment. For genome-wide association, which we think is the most likely use of the outbreds, choice will be guided by the genetic characterization provided here, most simply summarized by low LD, coupled with high mean minor allele frequency. Depending on the phenotype, an additional criterion may be the likelihood that heritable variation is present in a given colony; this could be determined either by family studies carried out with animals from the colony, or by determining whether strains contributing to the colony show phenotypic differences from published data, for example from the phenome project [37]. Our work, demonstrating the utility of the outbreds, is a starting place for ranking colonies according to their utility for genetic mapping. As costs fall, we anticipate that detailed characterization based on genomic sequence will become available and permit informed choices on the use of the colonies for genetic studies of complex traits in mice.

## Materials and Methods

### Sample collection and DNA extraction

We contacted ten commercial providers of outbred strain of mice, including Harlan Sprague Dawley (Hsd), Charles River Laboratories (Crl), Taconic Farms (Tac), Centre d'Elevage R. Janvier (Rj), Ace Animal (Aai), B&K Universal (Bk), Hilltop Laboratory Animals (Hla), Research and Consulting Company (Rcc), Scanbur (Sca), Simonsen Laboratories (Sim), and we collected on average 48 tail samples from unrelated mice from each colony (Table 1), representing 90% of all commercially available colonies of outbred mice. 48 unrelated individuals from six colonies were resampled at least one year after the initial collection. We also collected samples from control populations: 109 Collaborative Cross (CC) mice provided by Fuad Iraqi (Tel-Aviv University), 96 DNA samples of wild mice caught in the vicinity of Tucson (Arizona) provided by Michael Nachman, 12 unrelated Heterogeneous Stock (HS) DNA samples from our laboratory and 94 inbred strains purchased from the Jackson Laboratory. DNA was extracted from tail snips using a Nucleopure Kit (Tepnel, UK). DNA quality and quantity was assessed using UV spectrophotometry (Nanodrop) and 0.8% agarose gel electrophoresis.

### Genotyping

We designed extension and amplification primers for 351 SNPs using SpectroDESIGNER. Oligonucleotides were synthesized at Metabion (Germany) (Table S1). We used the Sequenom MassARRAY platform for genotyping these 351 SNPs over 4,000 DNA samples and SpectroTYPER Version 4.1 for data analysis. The resulting genotypes were then uploaded into an Integrated Genotyping System (IGS) [38]. We also obtained genome-wide SNPs genotyping data for six colonies using Affymetrix arrays. Three populations were genotyped using the 600K Affymetrix Mouse Diversity Array [16]. Three more populations were analysed using a precursor to this array, a gift from Mark Daly. DNA was prepared, hybridized and genotypes obtained following the manufacturer's protocol.

### Analyses of genetic relatedness

Data were stored in a relational database designed to manage genotypes and phenotypes [38]. Analyses were run either using software from the authors of each test or were implemented in R [39]. We tested Hardy-Weinberg Equilibrium (HWE) by the exact test [11] for all populations separately. Heterozygosity for each marker was calculated using PLINK [12]. We inferred individual ancestry proportions using a maximum likelihood method [24] in the program FRAPPE (http://www.fhcrc.org/labs/tang/). We used parameters described in [23], running the program for 10,000 iterations, with pre-specified cluster numbers, from K = 2 to 12. We found that independent runs yielded consistent results, with few additional clusters emerging after K = 9. However, it should be noted that given the small set of markers and the inclusion of markers in LD, our estimates of ancestry are likely to be biased. Fst for all pairs of populations was calculated using the FDIST2 program [40], [41] (http://www.rubic.rdg.ac.uk/~mab/software.html). An identity-by-state (IBS) matrix for all individuals was calculated using PLINK [12]. Principal component analysis was carried out using this IBS matrix. Genetic relationships were represented as a tree using agglomerative clustering implemented in R [39]. Haplotype blocks were estimated using PLINK [12] which implements the block finding algorithm found in HAPLOVIEW [42].

### Big dye sequencing

We used Primer3 to design oligonucleotide primers and carried out PCR reactions with Hotstar Taq obtained from Qiagen. Each 50 µl PCR contained 50 ng of genomic DNA, 1 Unit of HotStar Taq, 5 pmol of forward and reverse primers (synthesized at MWG Biotech, Ebersberg, Germany), 2 mM of each dNTP, 1× HotStar Taq PCR buffer as supplied by the enzyme manufacturer (contains 1.5 mM MgCl_2_, Tris-Cl, KCl and (NH_4_)_2_SO_4_, pH 8.7) and 25 mM MgCl_2_ (Qiagen). We ran the PCR reactions using a Touchdown (TD) approach. The temperature profile consisted of an initial enzyme activation at 95°C for 15 min, followed firstly by 13 cycles of 95°C for 30 sec, 64°C for 30 sec and 72°C for 60 sec, secondly by 29 cycles of 95°C for 30 sec, 57°C for 30 sec and 72°C for 60 sec, and finally by an incubation at 72°C for 7 min. PCR products were purified in a 96-well Millipore purification plate and resuspended in 30 µl of H_2_O. Two sequencing reactions were prepared for each DNA sample, one with the forward primer and one with the reverse primer using 50 ng DNA. The sequencing reaction consisted of an initial denaturation stage at 95°C for 1 min, followed by 29 cycles of 95°C for 10 sec, 50°C for 10 sec and 60°C for 4 mins. The PCR reagents were then removed from solution by an ethanol precipitation in the presence of sodium acetate. All sequencing reactions were run out on an ABI3700 sequencer and assembled by using phred/phrap [43]. Consed was then used for editing and visualisation of the assembly [44].

### Short read sequencing

The libraries were prepared from 3–5 µg sample genomic DNA following the Illumina standard genomic library protocol up to the ligation step, where a modified adapter was used. The resulting constructs were digested overnight at 37°C with 20 units high-concentration HindIII restriction enzyme (New England Biolabs) in a volume of 50 µl. The digested libraries were purified on Qiagen MinElute columns. A complementary biotinylated adapter was ligated to the sticky ends before selecting the fragments of 200 to 500 bp on a 2% agarose gel. The constructs with a HindIII-specific adapter were purified using Streptavidin magnetic beads (Invitrogen) following the manufacturer's instructions. The beads were finally resuspended in 25µl 10mM Tris pH8, of which 12.5 µl were used for the final PCR amplification (15 cycles) using specific amplification primers and Phusion DNA polymerase (Finnzymes). The resulting libraries were verified by TOPO cloning and sequencing before running them on an Illumina Genome Analyzer IIx for 38 cycles.

Libraries of reduced complexity for SNP discovery were made from pooled DNA samples. Genomic DNA was subject to complete HindIII restriction enzyme digestion and ligation to linkers. Libraries were then sequenced on an Illumina Genome Analyzer IIx. Since it has been shown that this method has a false SNP discovery rate of about 8% [22], we used three additional filtering criteria to increase confidence in SNP calls. First, following reports that SNPs falling at the ends of reads were unreliable, SNPs within three bases of the end of a read were discarded [22]. Second, SNPs that did not map to within 32 bases of a known HindIII restriction site were also discarded. Third, SNP detection is affected by read depth: where the coverage is less than fivefold, the proportion of novel SNPs rises to over 10%. We only report SNPs where the coverage is greater than tenfold.

### Phenotyping

We analysed 200 animals from three colonies: Crl:CFW(SW)-US_P08, HsdWin:CFW-NL and HsdWin:NMRI-NL. Blood samples were taken from a tail vein and we performed assays for serum alkaline phosphatase (ALP), ratio of CD4^+^ to CD8^+^ T-cells, concentration of high-density lipoproteins (HDL) in serum and mean red cell volume using published protocols [45].

### Genetic mapping

Where necessary, phenotypes were transformed into Gaussian deviates. Covariates (such as gender, age, experimenter, time) that explain a significant fraction of each phenotype's variance with ANOVA P-value<0.01 were included in subsequent statistical analyses. We use two mapping methods: a single point analysis of variance of each marker and a multi-point method. The single point method was implemented using linear modeling in R; the multipoint method is implemented in the R package HAPPY [17]. Region-wide significance levels are estimated by permuting the transformed phenotype values 1,000 times.

## Supporting Information

Figure S1 Multi-dimensional scaling of identity by state pairwise distances for all colonies, calculated using PLINK. The figure shows a reduced representation of the results, plotting the position on the first dimension (horizontal axis) against position on the second dimension (vertical axis).
(0.37 MB PDF)

Figure S2 PCA and multi-dimensional scaling of identity by state pairwise distances, calculated using PLINK. The figure shows a reduced representation of the results, plotting the position on the first dimension (horizontal axis) against position on the second dimension (vertical axis).
(0.37 MB PDF)

Table S1 SNPs used for genotyping the outbred mice
(0.05 MB XLS)

Table S2 Sequences variants found in outbreds
(0.22 MB XLS)

Text S1 Origins of commercial outbreds.
(0.07 MB DOC)
