# Supplementary material for: Commercially Available Outbred Mice for Genome-Wide Association Studies
Source: PLoS Genet. 2010 Sep 2;6(9):e1001085. doi: 10.1371/journal.pgen.1001085 (PMC2932682; doi:10.1371/journal.pgen.1001085)
Supplement: Figure S1 — Multi-dimensional scaling of identity by state pairwise distances for all colonies, calculated using PLINK. The figure shows a reduced representation of the results, plotting the position on the first dimension (horizontal axis) against position on the second dimension (vertical axis). (0.37 MB PDF) [file pgen.1001085.s001.pdf]

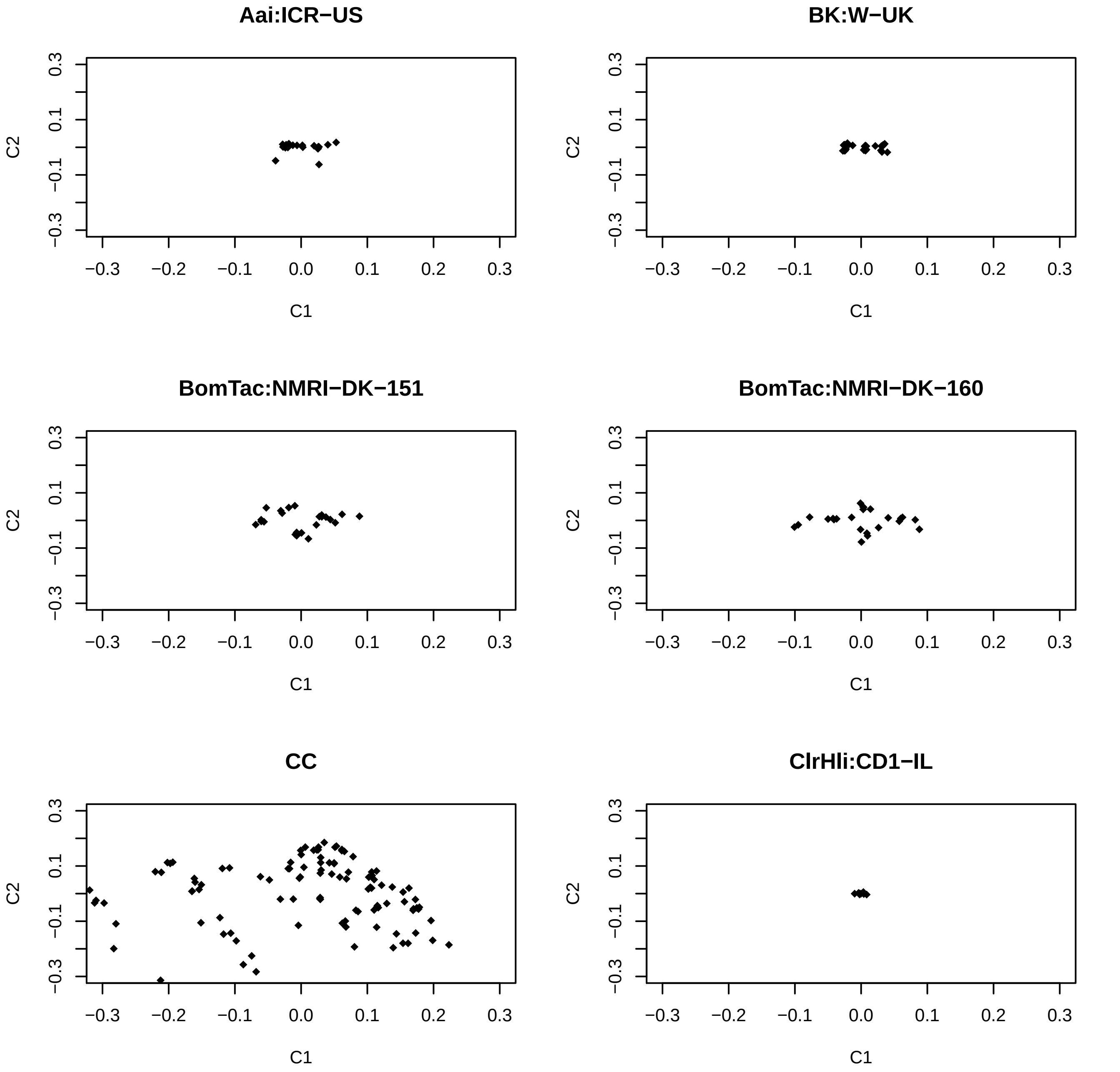
Aai:ICR−US
BK:W−UK
BomTac:NMRI−DK−151
BomTac:NMRI−DK−160
CC
ClrHli:CD1−IL
C1
C2
−0.3
−0.2
−0.1
0.0
0.1
0.2
0.3

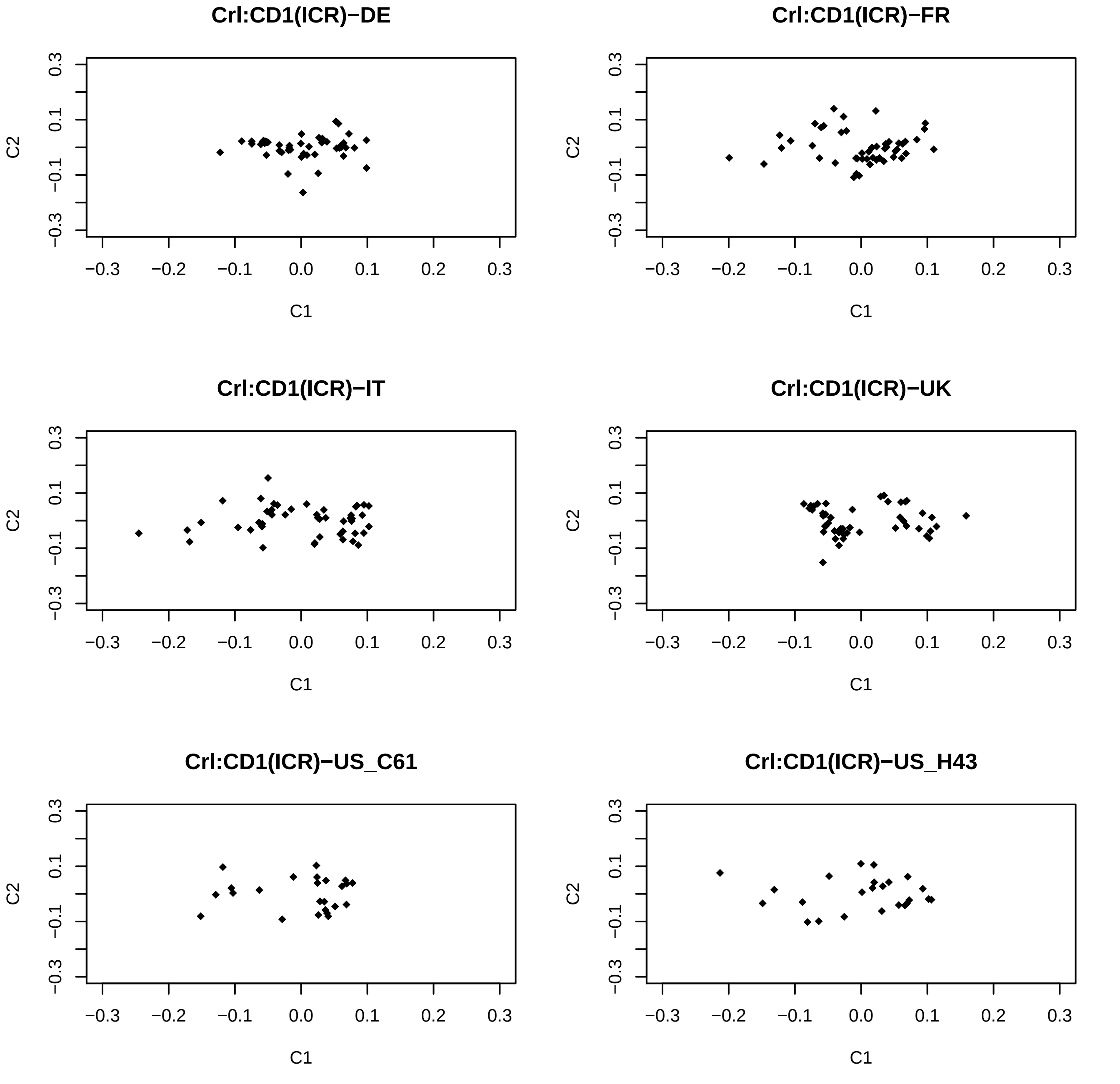
Crl:CD1(ICR)−DE
Crl:CD1(ICR)−FR
Crl:CD1(ICR)−IT
Crl:CD1(ICR)−UK
Crl:CD1(ICR)−US_C61
Crl:CD1(ICR)−US_H43
C1
C2
−0.3 −0.2 −0.1 0.0 0.1 0.2 0.3
−0.3 −0.1 0.1 0.3

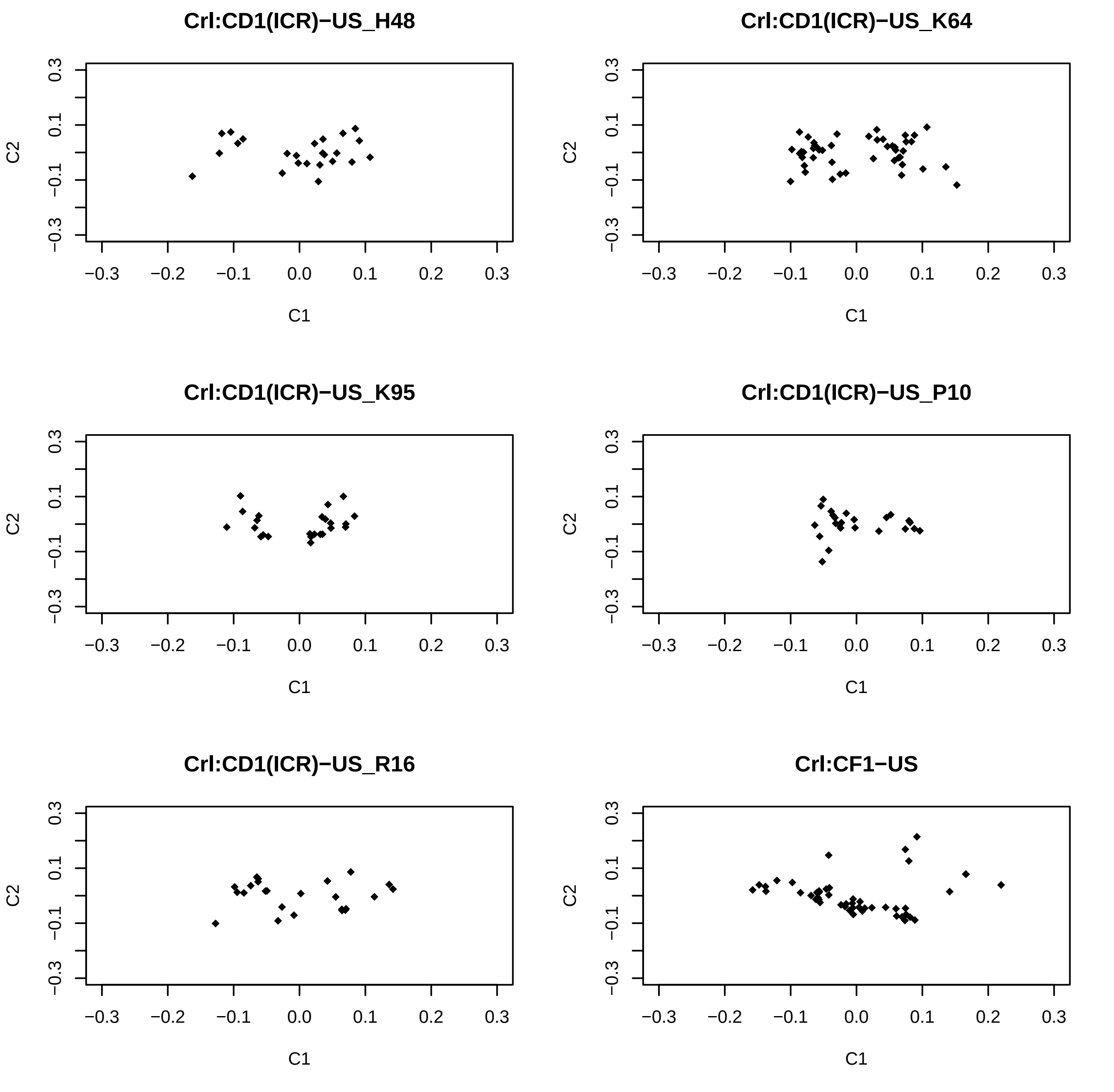
Crl:CD1(ICR)−US_H48
Crl:CD1(ICR)−US_K64
Crl:CD1(ICR)−US_K95
Crl:CD1(ICR)−US_P10
Crl:CD1(ICR)−US_R16
Crl:CF1−US
C1
C2
−0.3
−0.2
−0.1
0.0
0.1
0.2
0.3

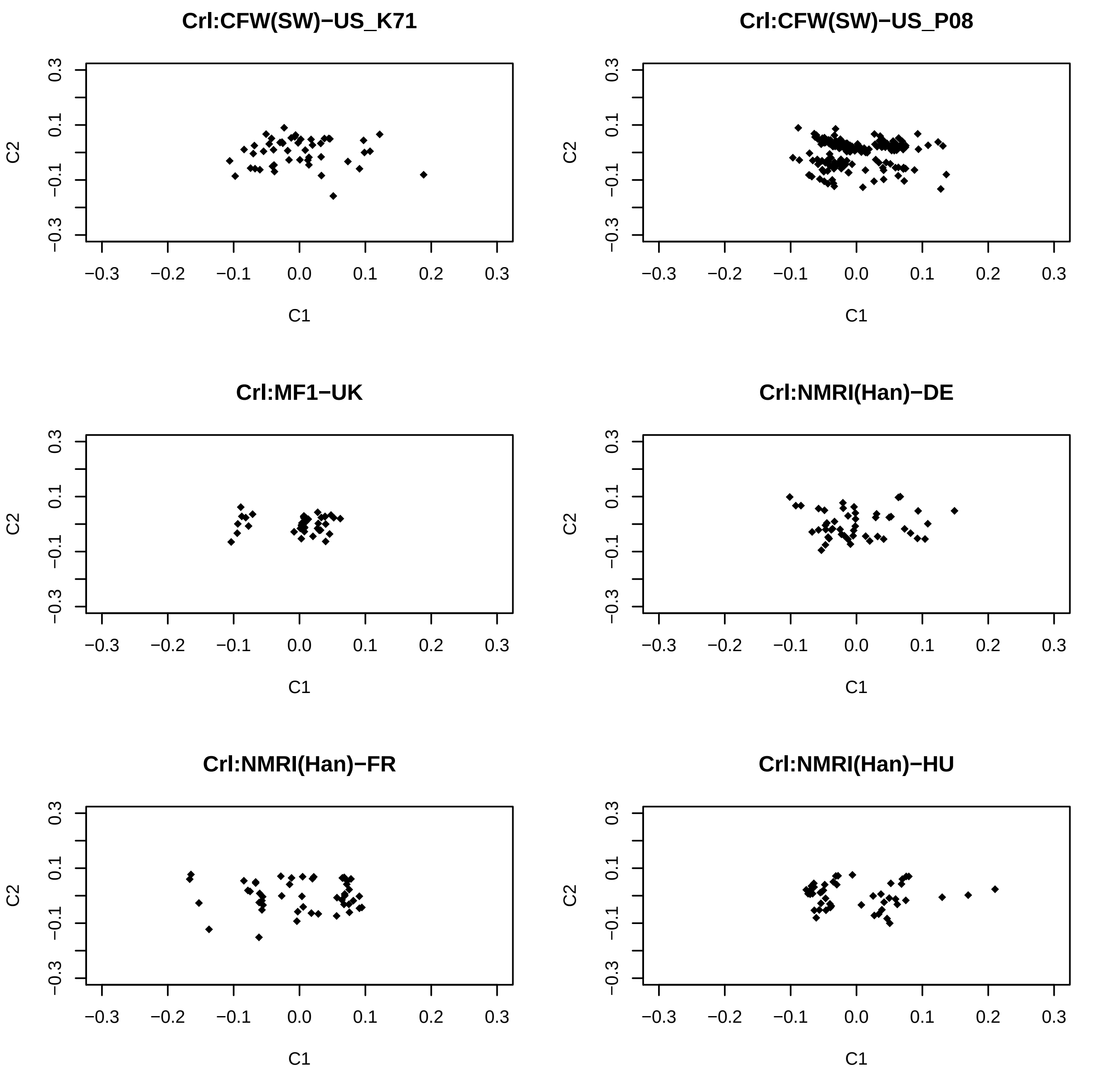
Crl:CFW(SW)−US_K71
Crl:CFW(SW)−US_P08
Crl:MF1−UK
Crl:NMRI(Han)−DE
Crl:NMRI(Han)−FR
Crl:NMRI(Han)−HU
C1
C2
−0.3
−0.2
−0.1
0.0
0.1
0.2
0.3

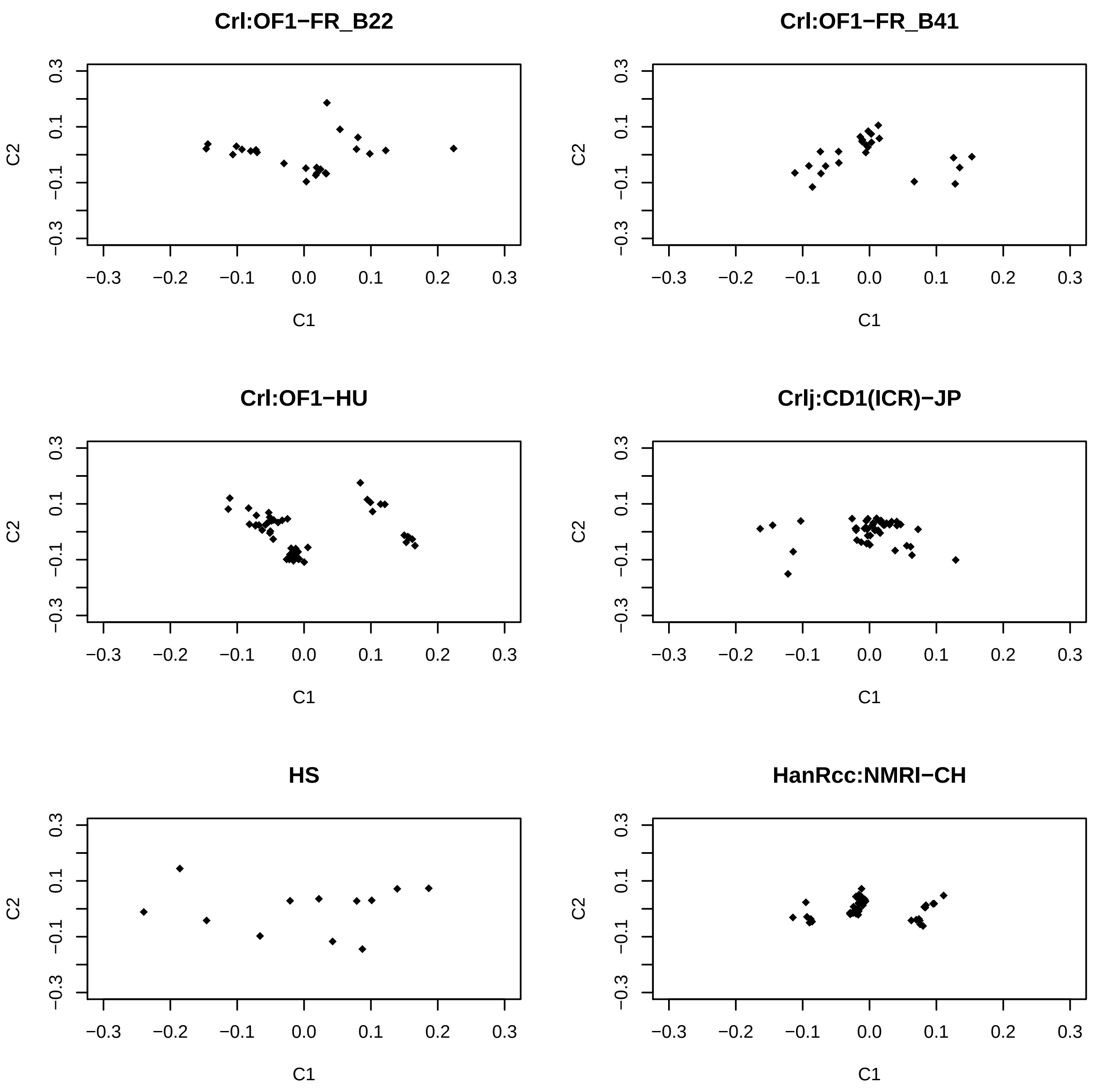
Crl:OF1−FR_B22
Crl:OF1−FR_B41
Crl:OF1−HU
Crlj:CD1(ICR)−JP
HS
HanRcc:NMRI−CH
C1
C2
−0.3 −0.2 −0.1 0.0 0.1 0.2 0.3
−0.3 −0.1 0.1 0.3

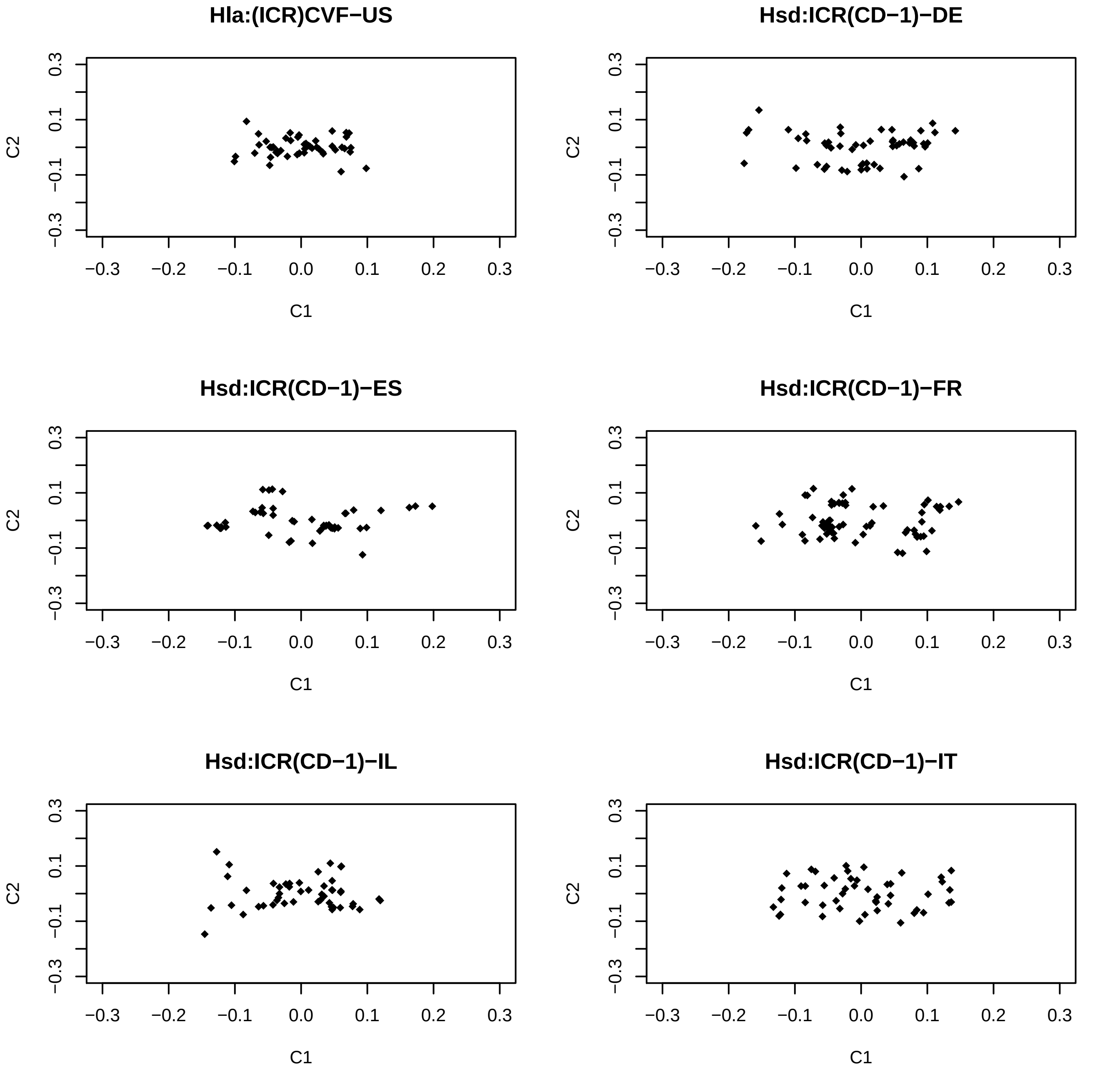
Hla:(ICR)CVF−US
Hsd:ICR(CD−1)−DE
Hsd:ICR(CD−1)−ES
Hsd:ICR(CD−1)−FR
Hsd:ICR(CD−1)−IL
Hsd:ICR(CD−1)−IT
C1
C2
−0.3 −0.2 −0.1 0.0 0.1 0.2 0.3
−0.3 −0.1 0.1 0.3

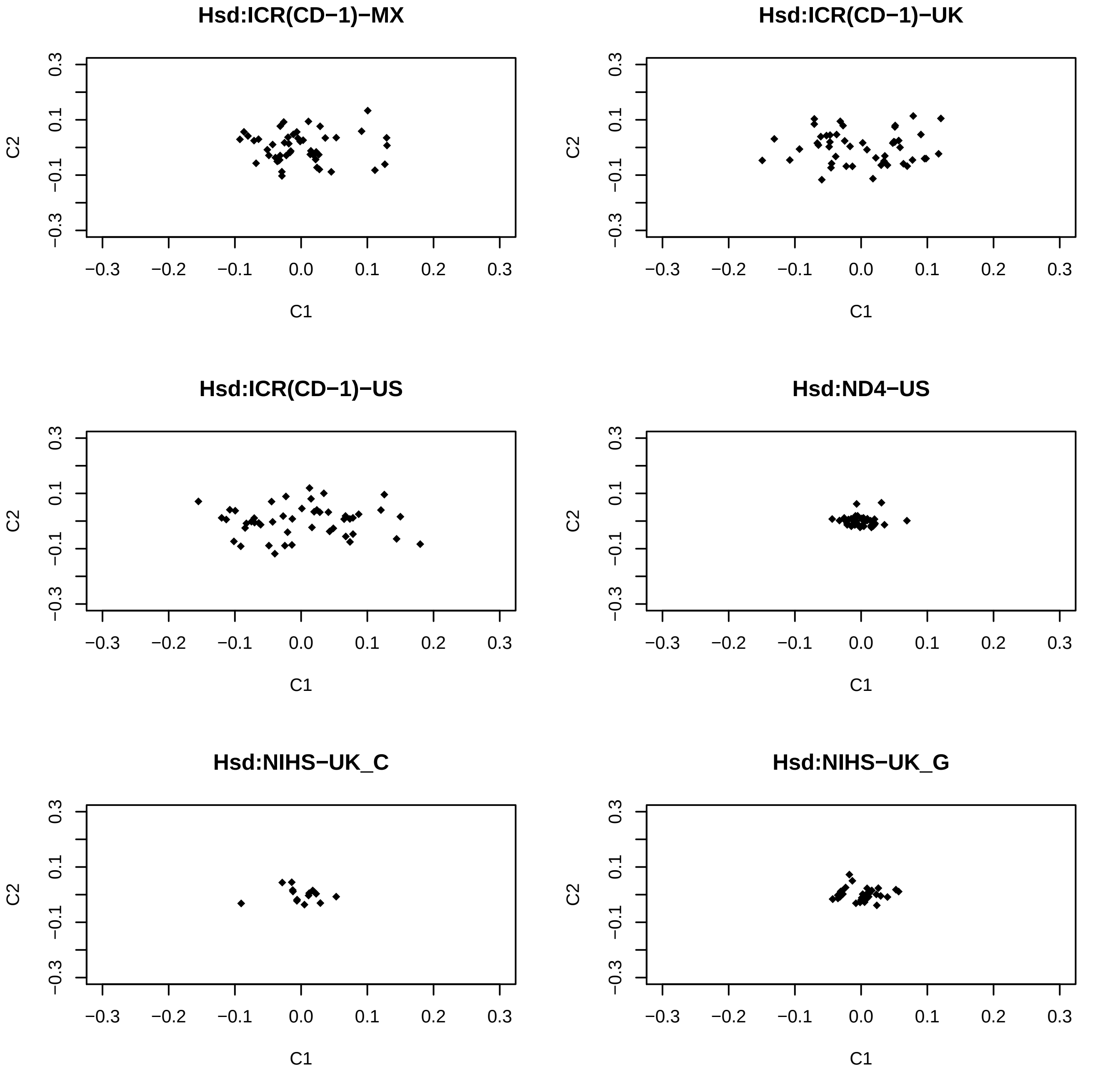
Hsd:ICR(CD−1)−MX
Hsd:ICR(CD−1)−UK
Hsd:ICR(CD−1)−US
Hsd:ND4−US
Hsd:NIHS−UK_C
Hsd:NIHS−UK_G
C1
C2
−0.3 −0.2 −0.1 0.0 0.1 0.2 0.3
−0.3 −0.1 0.1 0.3

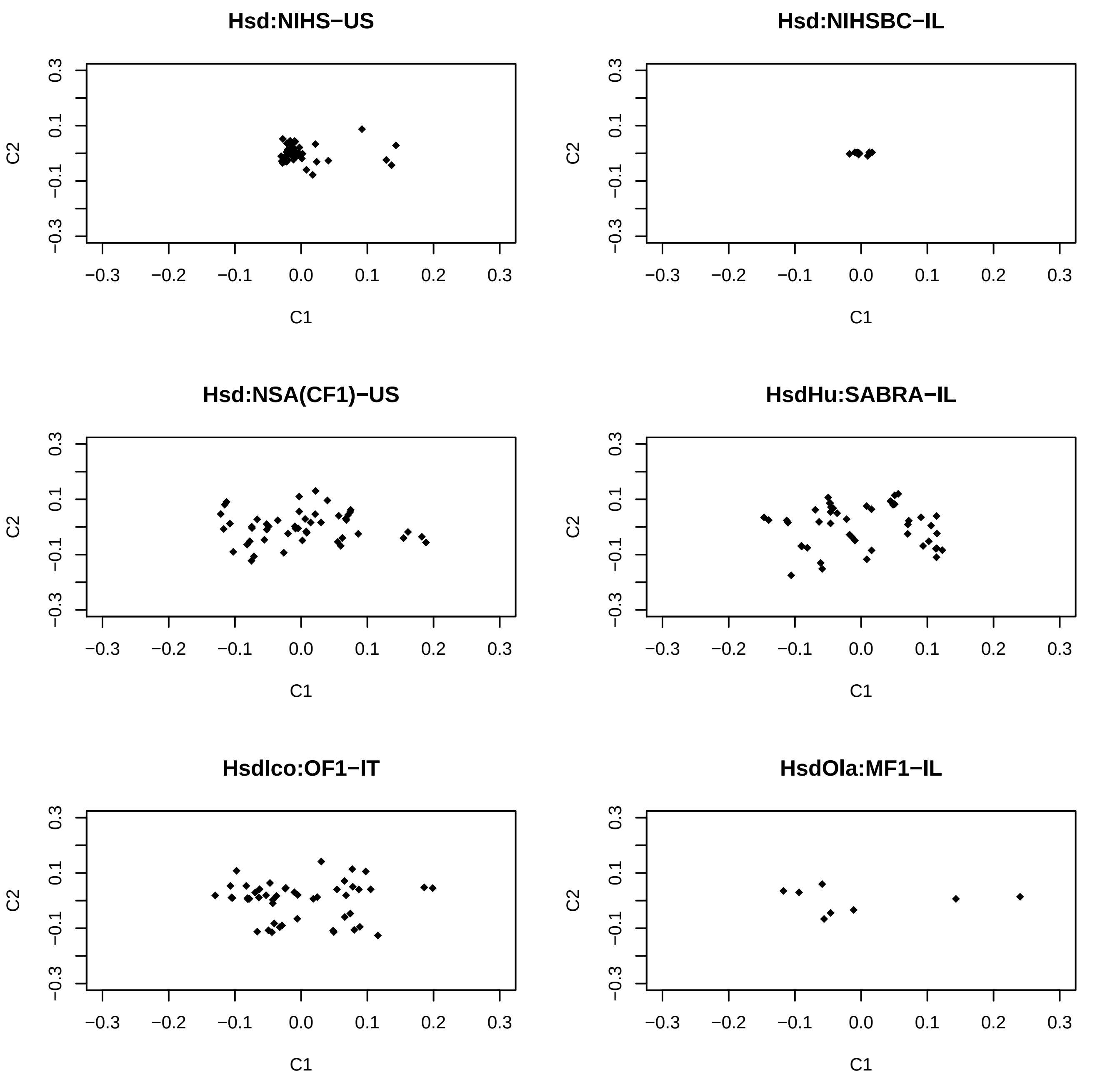
Hsd:NIHS−US
Hsd:NIHSBC−IL
Hsd:NSA(CF1)−US
HsdHu:SABRA−IL
HsdIco:OF1−IT
HsdOla:MF1−IL
C1
C2
−0.3
−0.2
−0.1
0.0
0.1
0.2
0.3

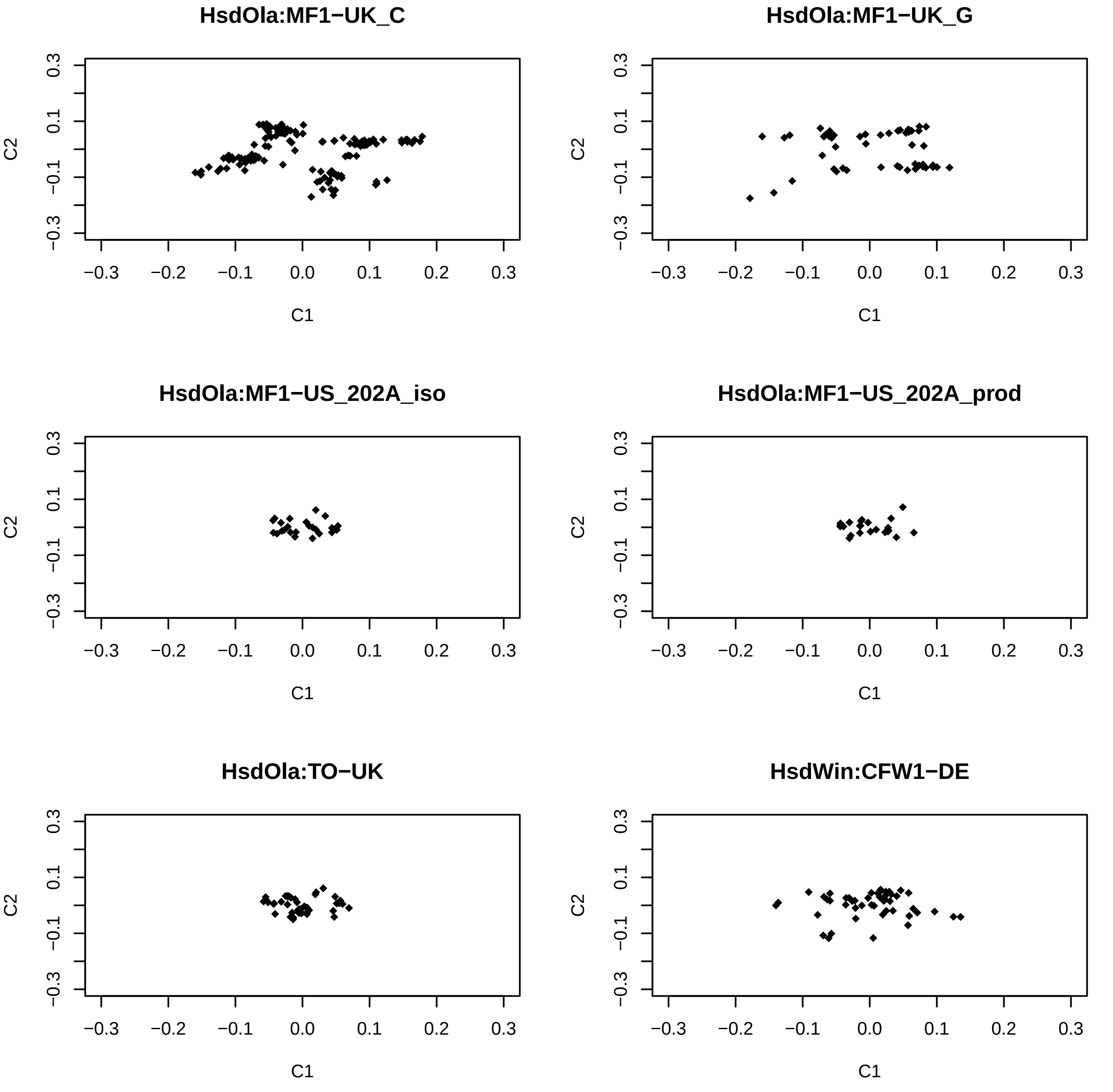
HsdOla:MF1−UK_C
HsdOla:MF1−UK_G
HsdOla:MF1−US_202A_iso
HsdOla:MF1−US_202A_prod
HsdOla:TO−UK
HsdWin:CFW1−DE
C1
C2
−0.3
−0.2
−0.1
0.0
0.1
0.2
0.3

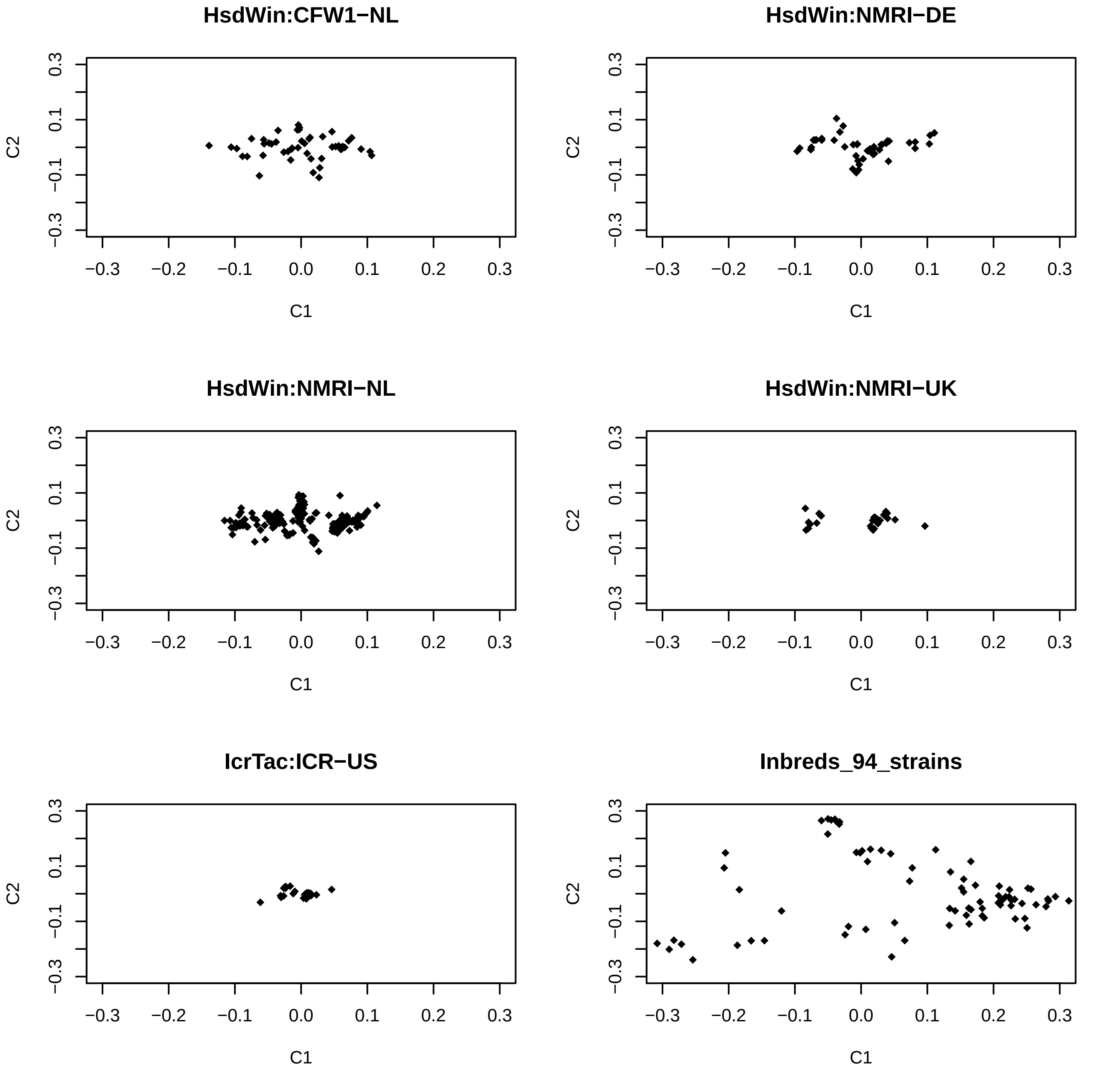
HsdWin:CFW1−NL
HsdWin:NMRI−DE
HsdWin:NMRI−NL
HsdWin:NMRI−UK
IcrTac:ICR−US
Inbreds_94_strains
C1
C2
−0.3
−0.2
−0.1
0.0
0.1
0.2
0.3

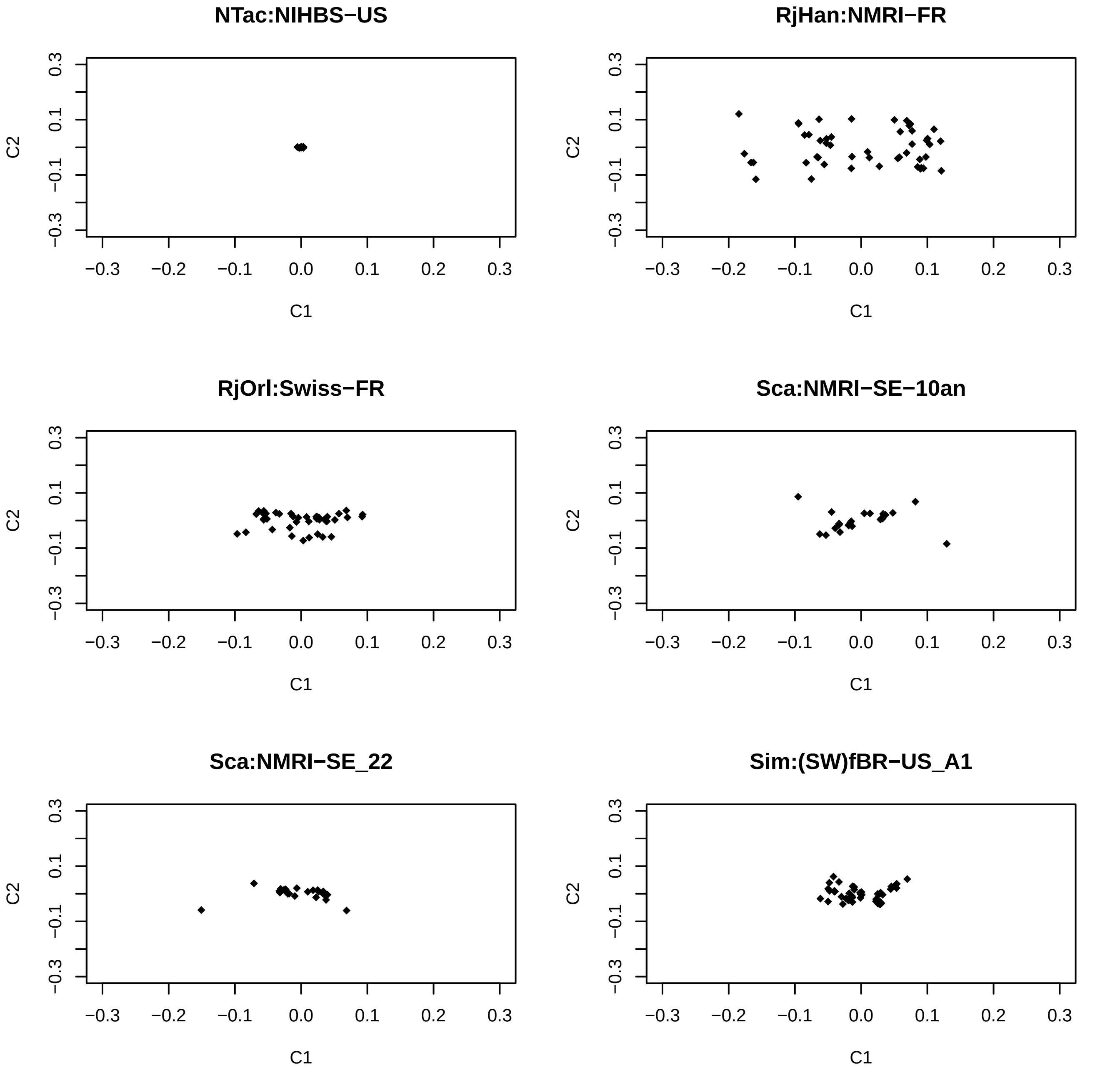
NTac:NIHBS−US
RjHan:NMRI−FR
RjOrl:Swiss−FR
Sca:NMRI−SE−10an
Sca:NMRI−SE_22
Sim:(SW)fBR−US_A1
C1
C2
−0.3
−0.2
−0.1
0.0
0.1
0.2
0.3

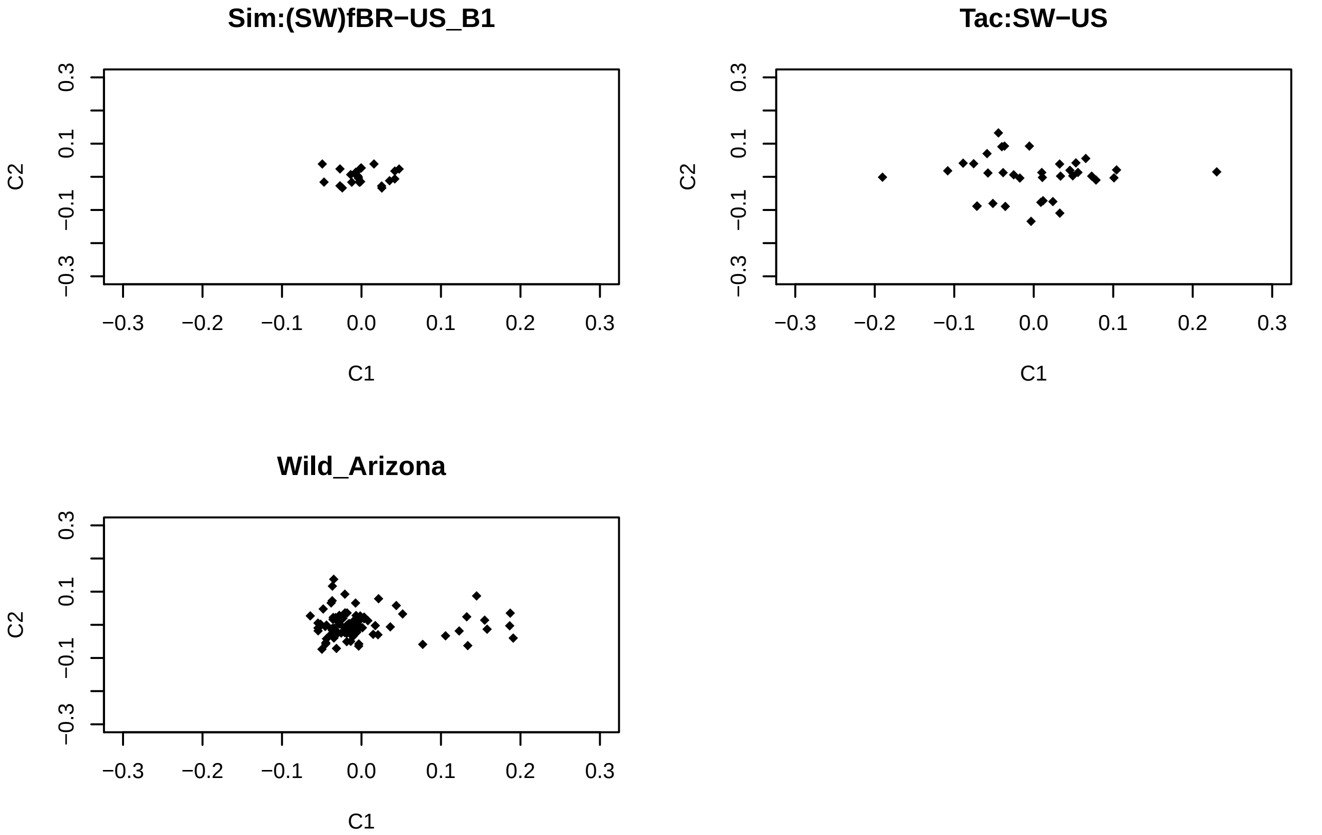

Sim:(SW)fBR−US_B1
Tac:SW−US
Wild_Arizona
C1
C2
−0.3
−0.2
−0.1
0.0
0.1
0.2
0.3
