# Supplementary material for: Commercially Available Outbred Mice for Genome-Wide Association Studies
Source: PLoS Genet. 2010 Sep 2;6(9):e1001085. doi: 10.1371/journal.pgen.1001085 (PMC2932682; doi:10.1371/journal.pgen.1001085)
Supplement: Text S1 — Origins of commercial outbreds. (0.07 MB DOC) [file pgen.1001085.s005.doc]

**SUPPLEMENTAL INFORMATION ON ORIGINS**

**Crl:NMRI(Han)**

Swiss-type mouse given by Clara Lynch to Poiley in 1937. He maintained an inbred line of these animals for 51 generations, before animals were transferred to the Naval Medical Research Institute. Introduced into Charles River Laboratories in 1979 from the Central Institute for Laboratory Animal Breeding Hannover (Germany). See <http://www.criver.com/>

**HsdWin:NMRI**

The original colony of Swiss mice in the US started from nine mice brought from Lausanne, Switzerland, in 1926 by Clara Lynch. In 1937 from Lynch to Poiley. Inbred by Poiley known as NIH/PI. At F51 to US Naval Medical Research Institute and known as NMRI. In 1955, to Bundes-Forschungsanstalt für Viruskrankheiten. In 1958, to Central Institute for Laboratory Breeding, Hannover. In 1981, from Central Institute for Laboratory Breeding, Hannover to Winkelmann. In 1998, from Harlan Winkelmann to Harlan Laboratories. See <http://www.harlan.com/>

**RjHan:NMRI** (Lucy Marie, personal communication)

Non-inbred Swiss mice from Lynch to Poiley, in 1937. Inbred by Poiley, known as NIH/P1, then to the US Naval Medical Research Institute. The Centre d’Elevage R. Janvier obtained mice from the Laboratory Animal Centre, Hannover, in 1982.

**HanRcc:NMRI** (Rosmarie Buser, personal communication)

It appears to be a SWISS-type mouse, which Clara Lynch gave to Poiley in 1937. He maintained an inbred line of this animal until the 51^st^ generation, before he transferred it to the Naval Medical Research Institute (NMRI). Over Zentralinstitut für Versuchstierzucht, Herrmann-Ehlers-Allee 57, D-3000 Hannover, this mouse was transferred to RCC, Ltd., Füllinsdorf (Switzerland) on March 7, 1989. A Poiley rotational breeding scheme with 12 groups using a 1M/1F ratio was used since then. RCC,Ltd. stopped producing this colony.

**Sca:NMRI**

The NMRI outbred model was developed by Lynch et al. Poiley of the National Institutes of Health received stock from Lynch in 1937. The mice were inbred as NIH/P1. The Naval Medical Research Institute (NMRI) received stock from Lynch. The Central Institute for Laboratory Animal Breeding, Hanover, Germany (Han) received stock from NMRI. The mice were random bred. Scanbur stock was obtained in 1994 (see <http://www.scanbur.eu/products/Lab_animals_NMRI.htm> ).

**HsdWin:CFW-1**

The original colony of Swiss mice in the US started from nine mice brought from Lausanne, Switzerland, in 1926 by Clara Lynch. In 1932, to LT Webster, Rockefeller Institute and in 1936, to Carworth Farms, New York, and named CF-1 (Carworth Farms strain 1). In 1968, from Carworth Farms to Winkelmann Versuchstierzucht GmbH & Co. In 1998 to Harlan Laboratories. See <http://www.harlan.com/>

**HsdOla:MF1** (personal communication)

In the early 1970s an outbred albino mouse stock maintained at various places was the LACA (Laboratory Animal Centre A-strain mice formally called CFW) standard outbred stock. The LACA outbred stock originated from Swiss mice (Lynch 1969). Around the same time, Carworth Farms Europe (part of Huntingdon Life Science) also produced an outbred albino called CS1(from Scientific Products Farm, Manston, Kent) mouse related to Swiss mice. The rodent production director at OLAC, Mike Flack, took both stocks of outbred animals, mixed them together, and called the mouse he had produced the MF1. Harlan Laboratories, Inc. received the animals in 1992. Also see <http://www.harlan.com/>

**Crl:MF1**

These albino mice, which were originally developed at OLAC prior to 1970, came from a cross between the Laboratory Animal Centre A-strain mice (‘LACA’ - but formerly called CFW) and the CS1 from Scientific Products Farm (acquired by Charles River Laboratories). The stock was rederived by Charles River Laboratories in 2004 and subsequently transferred to Charles River Laboratories UK in April 2005. See <http://www.criver.com/>

**Crl:CFW(SW)**

This stock resulted from the selective inbreeding by Dr. Leslie Webster using foundation animals from a large colony of Swiss mice maintained at Rockefeller Institute following importation from Switzerland in 1926. To Carworth Farms from Rockefeller Institute. Highly inbred at the time they were acquired by Carworth. This line was reduced to a single pair and progeny outbred from that point forward to form a new stock. To Charles River in 1974 from a representative cross section of the Carworth CFW colony. See <http://www.criver.com/>

**Crl:CD1(ICR)**

The original group of Swiss mice that served as progenitors of this stock consisted of 2 male and 7 female albino mice derived from a non-inbred stock in the laboratory of Dr. de Coulon, Centre Anticancéreux Romand, Lausanne, Switzerland. These animals were imported into the United States by Dr. Clara Lynch of the Rockefeller Institute in 1926.

The Hauschka (Ha/ICR) stock was initiated in 1948 at the Institute for Cancer Research in Philadelphia from “Swiss” mice of Rockefeller origin. From the ICR, they were transferred to Dr. Edward Mirand at Roswell Park Memorial Institute where they were designated as HaM/ICR. In 1959, they were transferred to Charles River Laboratories

and hysterectomy rederived the same year. See <http://www.criver.com/>

**Hla:(ICR)CVF**

The mice originated from animals at the Centre Anticancereus Romand in Switzerland. From their origin in Switzerland, they became known as “Swiss.” The mice were then imported to the Rockefeller Institute in 1926. From Rockefeller Institute, the animals were passed to the Institute for Cancer Research in 1948. At this point, the mice derived the designation ICR. From the Institute of Cancer Research, the mice then went to Roswell Park Memorial Institute and then to Charles River Laboratories, Inc. in 1959. In the 1970’s, the mice were obtained by I.C.I. Americas, Inc. and rederived at Hilltop Lab Animals, Inc. In 2002, the mice were rederived by cesarean section. See <http://hilltoplabs.com/>

**BK:W** (Peter Cook, personal communication)

This line has been bred at Grimston since before 1977 (precise date not recorded in source used). It was developed from albino outbred mice obtained from British Drug Houses (BDH), Imperial Chemical Industries (ICI) and Schofield. The mice from the three sources were all CD-1, i.e. they originated from the caesarean derivation (CD) of a non-inbred Swiss albino mouse in 1959. The CD-1 designation is that of Charles River. BK:W designation is used as a derivation of the CD-1 achieved through obtaining stock from these three sources. Currently, this colony is maintained using a 1M/6F ratio in a Poiley rotational scheme with three groups.

**Hsd:ICR(CD-1)**

The Hsd:ICR (CD-1) mouse model is of Swiss origin and descended from the original two male and seven female albino non-inbred mice, imported by the Rockefeller Institute in 1926 from Lausanne, Switzerland. Descendants were distributed worldwide and the Ha/ICR was established in 1948 at the Institute of Cancer Research (ICR). A portion of the Ha/ICR colony was given to the Roswell Park Memorial Institute (RPMI). Charles River Laboratories (CRL) received their nucleus from RPMI in 1959, and caesarean rederived. Harlan Laboratories obtained breeding stock from Charles River Laboratories, Wilmington, Massachusetts in 1983. See <http://www.harlan.com/>

**ClrHli:CD-1** (Miki Amit, personal communication)

Harlan Israel obtained CD-1 mice from the Weizmann Institute in 1993 and in 2004. The colony started with 16 breeding females.

**IcrTac:ICR**

The ICR outbred model was developed by Dr. T. S. Hauschka of Fox Chase Cancer Center in 1948. Dr. Hauschka left Fox Chase a portion of his colony. Taconic received breeder stock from Fox Chase in 1993. The mice were derived by hysterectomy into IBU colonies and are maintained by Poiley rotational bred mice with six groups and 1/3 M/F ratio. See <http://www.taconic.com/>

**Aai:ICR** (Denise Chroscinski, personal communication)

Ace Animals, Inc. bought ICR mice from Taconic in June 1994 and since maintained them using a 1M/2F ratio in a circular pair rotational breeding scheme with 10 groups. Colony size has increased to 2000 from 2007 to 2010.

**Hsd:NIHS**

The original colony of Swiss mice in the US started from nine mice brought from Lausanne, Switzerland, in 1926 by Clara Lynch. In 1932, to LT Webster, Rockefeller Institute. In 1935, to the National Institutes of Health, Bethesda, and these mice were the founders of the general purpose colony N:GP(S). In 1936, the N:NIH(S) was derived from this colony. From National Institutes of Health to Harlan Laboratories in 1987 (Stephen Hillen, personal communiation).

**NTac:NIHBS**

The Black Swiss outbred model was developed by Dr. Carl Hansen of the NIH Genetic Resource. N:NIH Swiss outbred mice were backcrossed to a C57BL/6N inbred background to generate a hybrid mouse heterozygous for the agouti locus. The mice were backcrossed ten generations (N10) to eliminate the agouti and albino alleles. Taconic received NTac:NIHBS litters in 1991. The mice were derived by caesarean in 1992 and rederived by embryo transfer in 2005. See <http://www.taconic.com/>

**Hsd:ND4**

The original colony of Swiss mice in the US started from nine mice brought from Lausanne, Switzerland , in 1926 by Clara Lynch. In 1932, to LT Webster, Rockefeller Institute. From Webster to the University of Notre Dame, Notre Dame, Indiana. From the University of Notre Dame to Harlan Laboratories. See <http://www.harlan.com/>

**Tac:SW**

Taconic received the Swiss Webster outbred model from the Rockefeller Institue through Rockland Farms Inc in 1940. The mice have been maintained as a closed colony since 1951. The mice were derived by caesarean from randomly chosen breeders and reintroduced into a Barrier Nucleus Expansion Colony in 1965-1969. The mice were derived by caesarean in 1983 from randomly chosen breeders from all Taconic's barrier units. See <http://www.taconic.com/>

**RjOrl:SWISS** (Lucy Marie, personal communication)

Selected by Carworth Farms in 1935, for its high performance of productivity. The Centre d’Elevage R. Janvier obtained mice from the Centre de Service des Animaux de Laboratoire (CSAL) in 1965.

**Sim:(SW)fBR**

Original stocks received from Rolfsmeyer Co. in 1956; crossed with NIH Swiss received from Ace Animals in 1981. Caesarean rederived in 1997. See <http://www.simlab.com/>

**Crl:OF1**

In 1935, Carworth Farm began to breed a line of mice for vigor and productivity. Progenitors originating from a colony bred in Missouri were bought and the strain was named CF1 (Carworth Farm 1). This strain was introduced to Charles River Laboratories France in 1967, and it acquired the name OF1 (Oncins France 1). See <http://www.criver.com/>

**HsdIco:OF1** (Val Summers, personal communication)

Harlan Italy obtained mice from Charles River Laboratory in 2004. In December 2009, there were 3012 breeding females in this colony. A random breeding scheme was in placed with two females for one male.

**Crl:CF-1**

Obtained by Carworth Farms, UK, from a Missouri laboratory. Not descended from “Swiss” mice from Rockefeller Institute (probably of wild albino origin).Founded in 1935 and intensively inbred by Carworth for over 20 generations. This line was then reduced to a single pair and progeny outbred from that point forward to form a new stock. To Charles River in 1974 from a representative cross section of the Carworth CF-1 colony. Also see <http://www.criver.com/>

**Hsd:NSA(CF1)**

The original breeding stock for this colony was obtained from a breeder whose stock has been used extensively in the early work with pneumococci. At Carworth Farms, this stock acquired the name CF No.1. From Charles River Laboratories, Wilmington, Massachusetts, to Harlan Laboratories. See <http://www.harlan.com/>

**HsdOla:TO**

Stock originally developed prior to 1940 by Dr M. Theiler, State Serum Institute, Denmark, for studies of virus infections causing encephalomyelitis in mice. In 1953, to National Institute of Medical Research, Mill Hill. To Clinical Research Centre, Harrow in 1970. From Clinical Research Centre, Harrow to OLAC (now Harlan Laboratories) in 1979. Maintained as a closed colony since. See <http://www.harlan.com/>

**HsdHu:SABRA** (Miki Amit, personal communication)

Harlan Israel obtained mice from the Authority for Animal Facilities of the Hebrew University, Jerusalem, Israel. Currently this colony is maintained by Harlan Israel. A random breeding scheme is in use with a ratio of 1M/2F. The number of breeding females in the colony is 100.
